# Functional genomics analysis identifies T and NK cell activation as a driver of epigenetic clock progression

**DOI:** 10.1186/s13059-021-02585-8

**Published:** 2022-01-14

**Authors:** Thomas H. Jonkman, Koen F. Dekkers, Roderick C. Slieker, Crystal D. Grant, M. Arfan Ikram, Marleen M. J. van Greevenbroek, Lude Franke, Jan H. Veldink, Dorret I. Boomsma, P. Eline Slagboom, B. I. O. S. Consortium, Bastiaan T. Heijmans

**Affiliations:** 1 Molecular Epidemiology, Department of Biomedical Data Sciences, Leiden University Medical Center, Einthovenweg 20, 2333 ZC Leiden, The Netherlands; 2 Department of Cell and Chemical Biology, Leiden University Medical Center, Einthovenweg 20, 2333 ZC Leiden, The Netherlands; 3 Department of Epidemiology and Biostatistics, Amsterdam Public Health Institute, Amsterdam Cardiovascular Sciences Institute, Amsterdam UMC, location VUmc, Amsterdam, The Netherlands; 4 Department of Epidemiology, Erasmus Medical Center, Doctor Molewaterplein 40, 3015 GD Rotterdam, The Netherlands; 5 Department of Internal Medicine and School for Cardiovascular Diseases, Maastricht University Medical Center, Universiteitssingel 50, 6229 ER Maastricht, The Netherlands; 6 Department of Genetics, University Medical Centre Groningen, Antonius Deusinglaan 1, 9713 AV Groningen, The Netherlands; 7 Department of Neurology, Brain Center Rudolf Magnus, University Medical Center Utrecht, Universiteitsweg 100, 3584 CG Utrecht, The Netherlands; 8 Department of Biological Psychology, Vrije Universiteit Amsterdam, Van der Boechorststraat 1, 1081 BT Amsterdam, The Netherlands

## Abstract

**Background:**

Epigenetic clocks use DNA methylation (DNAm) levels of specific sets of CpG dinucleotides to accurately predict individual chronological age. A popular application of these clocks is to explore whether the deviation of predicted age from chronological age is associated with disease phenotypes, where this deviation is interpreted as a potential biomarker of biological age. This wide application, however, contrasts with the limited insight in the processes that may drive the running of epigenetic clocks.

**Results:**

We perform a functional genomics analysis on four epigenetic clocks, including Hannum’s blood predictor and Horvath’s multi-tissue predictor, using blood DNA methylome and transcriptome data from 3132 individuals. The four clocks result in similar predictions of individual chronological age, and their constituting CpGs are correlated in DNAm level and are enriched for similar histone modifications and chromatin states. Interestingly, DNAm levels of CpGs from the clocks are commonly associated with gene expression *in trans*. The gene sets involved are highly overlapping and enriched for T cell processes. Further analysis of the transcriptome and methylome of sorted blood cell types identifies differences in DNAm between naive and activated T and NK cells as a probable contributor to the clocks. Indeed, within the same donor, the four epigenetic clocks predict naive cells to be up to 40 years younger than activated cells.

**Conclusions:**

The ability of epigenetic clocks to predict chronological age involves their ability to detect changes in proportions of naive and activated immune blood cells, an established feature of immuno-senescence. This finding may contribute to the interpretation of associations between clock-derived measures and age-related health outcomes.

**Supplementary Information:**

The online version contains supplementary material available at 10.1186/s13059-021-02585-8.

## Background

Epigenetic clocks are sets of CpG dinucleotides whose DNA methylation (DNAm) can be used to accurately predict a person’s chronological age [1]. In recent years, various epigenetic clocks have been developed [2–5]. Well-known examples are the clocks developed by Hannum et al., trained on blood samples and containing 71 CpGs [2], and Horvath, a multi-tissue predictor consisting of 353 CpGs [3]. A popular application of such clocks is to calculate the deviation of predicted age from chronological age in a population and test for its association with a broad range of age-related health outcomes under the assumption that the deviation is a biomarker of biological age [6, 7]. The wide application of these clocks, however, contrasts with the lack of insight into why they are accurate predictors of chronological age.

Various mechanisms have been considered to underlie progression of epigenetic clocks. The chronological age as predicted by Horvath’s clock [3] was shown to be independent from the accumulation of senescent cells [8] and the progression this clock has been hypothesized to be due to a gradual decline in epigenetic control [9]. A key question is to what extent the clocks measure intrinsic cellular processes or extrinsic processes, in particular age-related shifts in proportions of certain cell types within a tissue. Recent work by Zhang et al. underlined the influence of cell-type proportions on clock performance and indicated that they underlie associations with age-related phenotypes [5]. Characterization of how epigenetic clocks and their constituting CpGs are associated with gene expression may shed more light on the biological processes involved.

To gain insight in the processes that contribute to epigenetic clock progression, we adopted a functional genomics approach in which we systematically evaluated the association between clock CpG DNAm and genome-wide gene expression as read-out of the biological changes involved in progression of the clocks. We included four established epigenetic clocks into our analyses, all of which use the DNAm of CpGs throughout the genome to predict chronological age (Table 1): the blood clock developed by Hannum et al. containing 71 CpGs (Hannum Bld) [2], the multi-tissue clock developed by Horvath consisting of 353 CpGs (Horvath MT) [3], the skin/blood clock developed by Horvath et al. including 391 CpGs (Horvath Skn/Bld) [4], and the blood/saliva clock developed by Zhang et al. spanning 514 CpGs (Zhang Bld/Slv) [5]. Our analyses included whole blood samples from 3132 individuals with DNAm and RNA-seq data, as well as public cell type-specific transcriptomic and methylomic data, and highlighted age-related changes in T and NK cell phenotypes as a driver of epigenetic clock progression.

**Table 1:** Description of the investigated epigenetic clocks. "Training samples" refers to the number of samples on which the clock was trained; "Training tissues" refers to the tissues used for development of the clock

Epigenetic clock	Number of CpGs	Training samples	Training tissues	Publication date
Hannum Bld	71	656	Whole blood	2013 [2]
Horvath MT	353	3931	27 tissue types	2013 [3]
Horvath Skn/Bld	391	896	Whole blood, buccal cells, various skin cells	2018 [4]
Zhang Bld/Slv	514	13566	Whole blood, saliva	2019 [5]

## Results

### Epigenetic clocks accurately predict chronological age and show high similarity

Our analyses were performed on whole blood samples from 3132 unrelated individuals, aged 18 to 87, originating from 6 Dutch cohorts (Table 2), for which both DNAm data and gene expression data were obtained, measured by Illumina 450K arrays and RNA-seq, respectively. Only samples for which both DNAm and gene expression data passed QC were analyzed.

**Table 2:** Characteristics of the 6 cohorts comprising our dataset

Cohort	Number of samples	Age (years)			Male/female percentage
		Mean	SD	Range	
CODAM	147	65.5	6.9	50-79	54%/46%
LL	656	46.4	13.6	18-81	43%/57%
LLS	626	58.3	6.8	30-79	48%/52%
NTR	854	37.8	14.8	18-79	34%/66%
PAN	155	67.7	9.5	37-87	61%/39%
RS	694	62.5	6.3	51-87	42%/58%
**Total**	**3132**	**52.5**	**16.2**	**18-87**	**43%/57%**

**Fig. 1:**
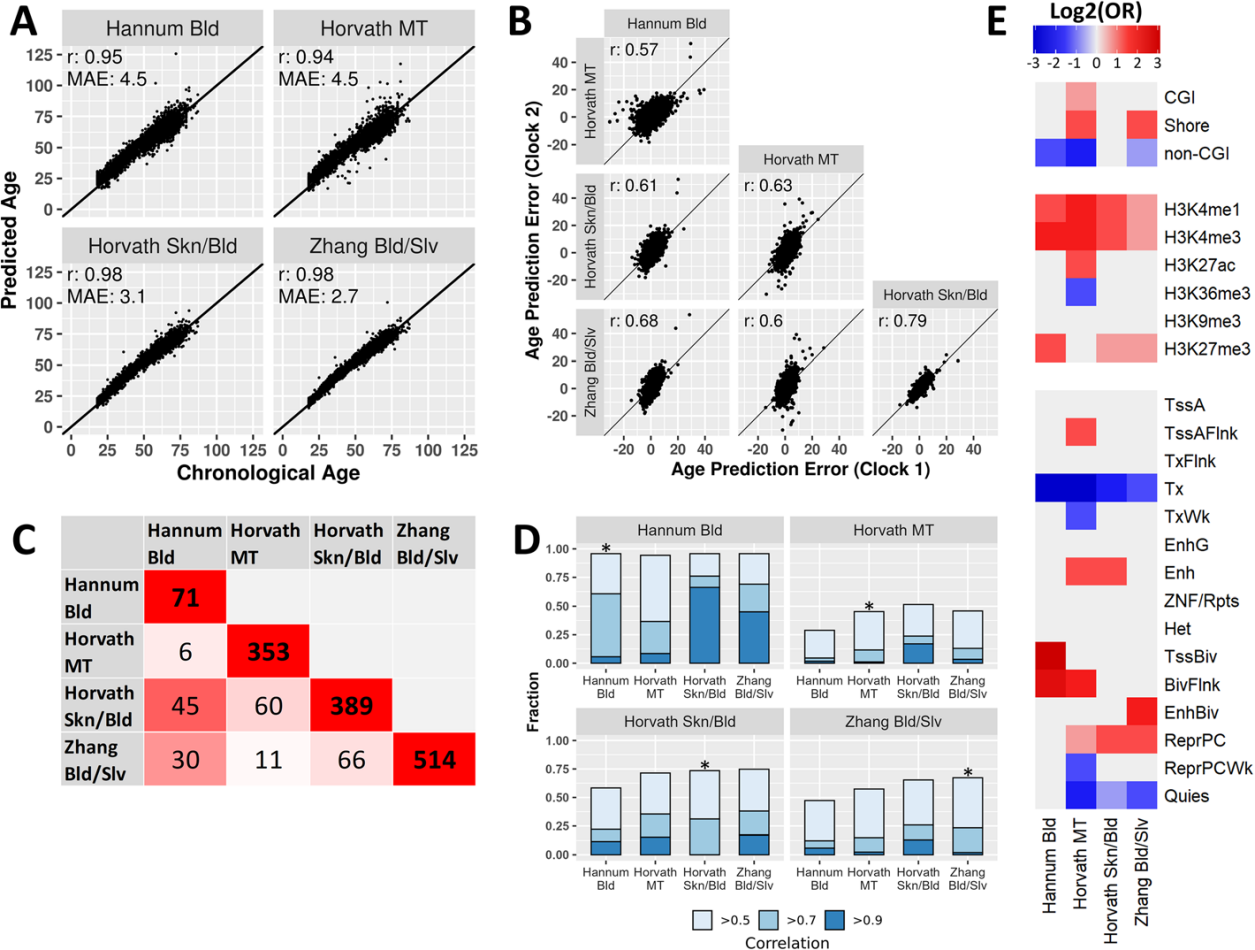
Characteristics of epigenetic clocks. **A** Prediction of age by the four epigenetic clocks. Prediction accuracy was analyzed using Pearson correlation (*r*) between chronological and predicted age, as well as mean absolute error (MAE) of the predicted ages. **B** Concordance of the age prediction errors of the epigenetic clocks, analyzed using Pearson correlation (*r*) between the prediction errors of each clock pair. **C** Overlap of the CpGs comprising the epigenetic clocks. The cells on the diagonal represent total clock size, while the other cells represent the number of CpGs shared between each clock pair. The color of each cell represents the percentage of the smaller CpG set that overlaps with the larger CpG set (0% = white, 100% = red). **D** Percentage of each clock’s CpGs which have a proxy (defined by absolute Pearson correlation above 0.5) within the same clock or in the 3 other clocks. Percentages with a proxy correlation of at least 0.5, 0.7, and 0.9 are plotted in stacked bar charts. Correlations within a clock are marked with an asterisk (*). **E** Enrichment of clock CpGs in CpG-island-centric features (top section), histone modifications (middle section), and chromatin states according to *ChromHmm* (bottom section). Abbreviations: *OR*: odds ratio; *CGI*: CpG island; *TssA*: Active transcription start site; *TssAFlnk*: flanking active transcription start site; *TxFlnk*: transcription at gene 5' and 3'; Tx: strong transcription; *TxWk*: weak transcription; *EnhG*: genic enhancers; *Enh*: enhancers; *ZNF/Rpts*: ZNF genes plus repeats; *Het*: heterochromatin; *TssBiv*: bivalent/poised transcription start site; *BivFlnk*: flanking bivalent transcription start site/enhancer; *EnhBiv*: bivalent enhancer; *ReprPC*: repressed polycomb; *ReprPCWk*: weak repressed polycomb; *Quies*: quiescent/low

First, we applied 4 epigenetic clocks (Table 1) to the DNAm data to predict age. All clocks accurately predicted age in our data. The Pearson correlation (r) between chronological age and predicted age was greater than 0.90 for all clocks, but there were differences in the prediction errors (Fig. 1A). Hannum Bld and Horvath MT showed the highest age prediction error (mean absolute error (MAE) = 4.5 years), followed by Horvath Skn/Bld (MAE = 3.1 years), and the prediction error was lowest for Zhang Bld/Slv (MAE = 2.7 years). We found that the errors in age prediction of the epigenetic clocks were highly correlated between clocks, with the pairwise correlation coefficients ranging from 0.57 to 0.79 (Fig. 1B). Thus, a person whose predicted age exceeds their chronological age according to one clock was likely to have a similar deviation according to another clock. However, this was not the case for extreme differences between predicted and chronological age, which were generally not reproduced between clocks (Additional file 1: Fig. S1A-B). For example, of the individuals for whom the prediction error of Hannum Bld was 10 years or higher, 32% had a prediction error above 10 years according to Horvath MT, and only 4% according to Zhang Bld/Slv (Additional file 1: Fig. S1A-B, top row). However, the individuals marked as extreme by Zhang Bld/Slv were more consistent with the other clocks, with up to 91% overlap (Additional file 1: Fig. S1A-B, bottom row). These findings indicate that extreme deviations between chronological and predicted age should be interpreted with caution.

To test the extent to which the clocks captured the same information, we compared the individual CpGs constituting the clocks. We found that 45 out of the 71 CpGs making up Hannum Bld were also present in Horvath Skn/Bld (63%), and 30 were also included in Zhang Bld/Slv (42%), indicating that a large portion of the information captured by Hannum Bld is also captured by Horvath Skn/Bld and Zhang Bld/Slv (Fig. 1C). The other clock pairs showed markedly lower percentages of overlapping CpGs (ranging from 3% to 17%; Fig. 1C).

Notably, the similar predictions of the 4 clocks may not only stem from CpGs shared between the clocks, but also from non-shared CpGs whose methylation levels are correlated. Therefore, we analyzed, per clock, whether each of its CpGs had at least one moderate, strong, or very strong proxy (defined as absolute correlation above 0.5, 0.7, and 0.9, respectively) in the 3 other clocks. The majority of clock CpGs had at least a moderate proxy in the other clocks (Fig. 1D); the percentages varied from 94% (Hannum Bld with Horvath Skn/Bld) to 45% (Horvath MT with Hannum Bld). Therefore, it is plausible that both overlapping CpGs and correlation between non-overlapping CpGs contributed to the similar performance of the 4 clocks in predicting chronological age. Interestingly, while all clocks were trained using elastic net, a penalized regression method to limit correlation between features in a predictor [2–5], we nevertheless observed fairly strong internal correlations within each clock, with 45 to 96% of the clocks’ CpGs having a moderate proxy within the same clock (Fig. 1D). This indicates that there is notable redundancy in the CpGs included in the clocks.

The similarity between CpGs across clocks was also evident from their genomic annotations (Fig. 1E). Although CpGs of different clocks showed inconsistent enrichments for CpG-island related features, all clocks were enriched for the histone modifications H3K4me1 and H3K4me3, which are associated with active enhancers and active promoters respectively [10]. All clocks except Horvath MT were also enriched for H3K27me3, which is a mark for polycomb repression [10]. The latter finding was confirmed by the analysis of chromatin states. All clocks were enriched for either polycomb-repressed chromatin (ReprPC) or one or multiple types of bivalent states (TssBiv, BivFlnk, EnhBiv). Finally, all clocks were depleted for actively transcribed chromatin states (Tx).

### Epigenetic clock CpGs are associated *in trans* with the expression of genes related to T cell activity

To gain more insight into the biological phenomena that are captured by the epigenetic clocks, we investigated how DNAm of the individual clock CpGs correlated with gene expression. We did this by performing a linear regression analysis to find the associations between clock CpG DNAm and expression of genes *in cis* and *in trans*.

**Fig. 2:**
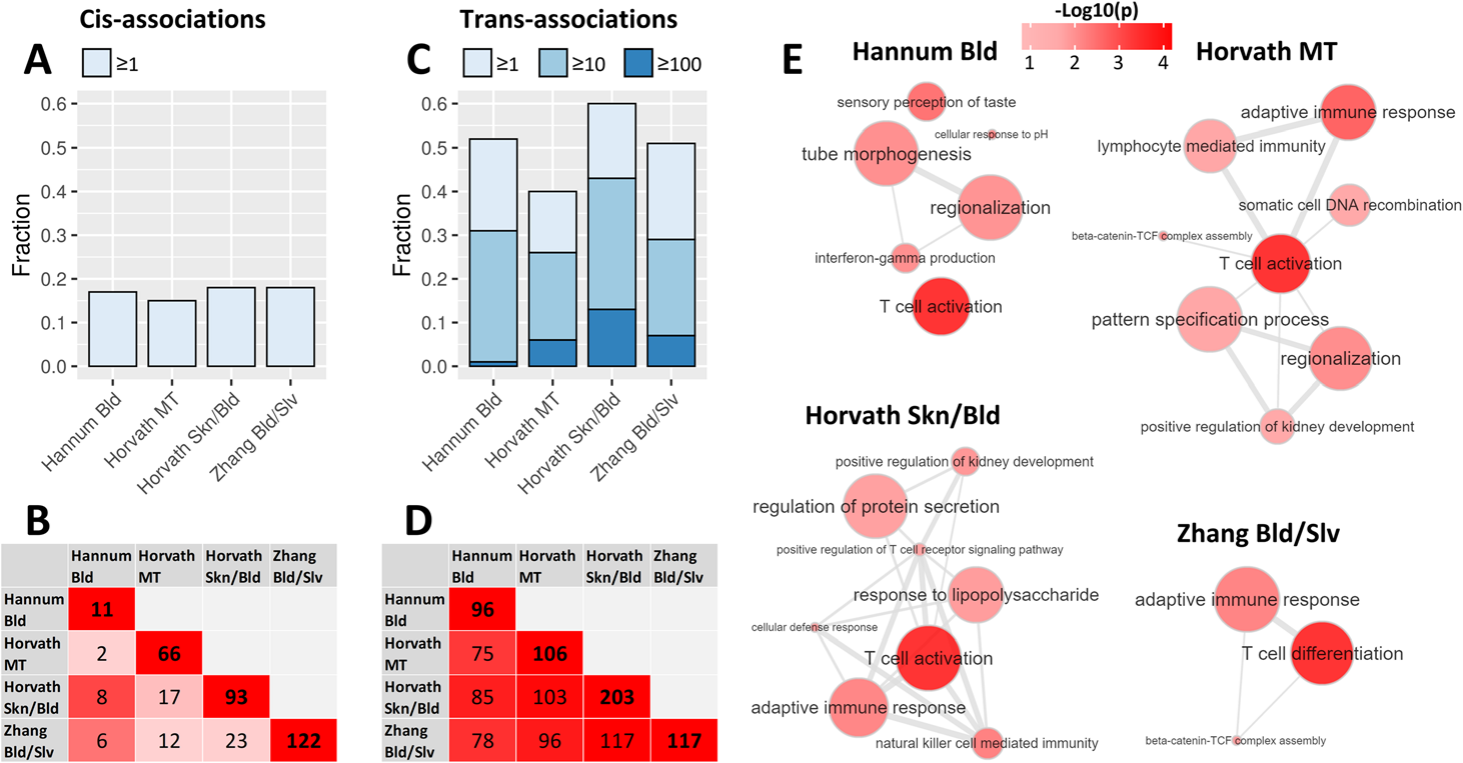
Associations between clock CpG DNAm and gene expression. **A** Percentages of CpGs belonging to the clocks which associate with the expression of at least 1 gene *in cis* (<100 kB distance). **B** Overlap of the genes associating with clock CpGs *in cis*. The cells on the diagonal represent the total number of genes associating with the CpGs of the clock *in cis*, while the other cells represent the number of *cis*-genes shared between each clock pair. The color of each cell represents the percentage of the smaller gene set that overlaps with the larger gene set (0% = white, 100% = red). **C** Percentages of CpGs belonging to the clocks which associate with the expression of at least 1, 10, or 100 genes *in trans* (>5 MB distance or different chromosomes). **D** Overlap of the genes associating with clock CpGs *in trans*. The cells on the diagonal represent the total number of genes associating with the CpGs of the clock *in trans*, while the other cells represent the number of *trans*-genes shared between each clock pair. The color of each cell represents the percentage of the smaller gene set that overlaps with the larger gene set (0% = white, 100% = red). Only *trans*-genes which were associated with at least 5% of any of the clocks were included. **E** Biological pathway gene ontology (GO) enrichments of *trans*-genes. Only *trans*-genes which were associated with at least 5% of any of the clocks were included. Networks were created using REVIGO, filtering out extremely similar GO-terms for clarity [11]. Nodes represent GO-terms, with node size depicting GO-term generality, and node color depicting enrichment *p* value. Highly related GO-terms which pass the similarity filter are connected by edges in the graph, with edge width representing term similarity

We first analyzed the associations *in cis* (<100 kB distance between CpG and gene). In each clock, 15–18% of CpGs associated with the expression of at least 1 gene *in cis*, with only minor differences between clocks (Fig. 2A; corrected for chronological age, blood cell counts, cohort, technical covariates, and latent factors). Across all clocks, 194 CpGs associated with the expression of 236 genes *in cis*. The *cis*-genes (Additional file 2: Table S1) followed similar trends to the CpGs shared between clocks, with a large percentage of the genes associating with Hannum Bld CpGs also associating with Horvath Skn/Bld or Zhang Bld/Slv CpGs, and other clock pairs showing moderate overlaps (Fig. 2B). For none of the clocks, the genes associating *in cis* were enriched for biological processes according to the gene ontology (GO) database, and no overarching patterns were identified upon manual inspection. Therefore, we concluded that the *cis*-genes had heterogeneous functions and did not involve distinct biological processes.

Next, we investigated the association between DNAm of clock CpGs and expression of genes *in trans* (>5 MB distance from CpG or located on a different chromosome). The clock CpGs associated with a markedly higher number of *trans*-genes (Additional file 2: Table S1), with 40–60% of each clock’s CpGs having a significant association (*p* < 10^-8^) with at least 1 gene *in trans* (Fig. 2C). A permutation test confirmed the statistical significance of association between CpGs and *trans*-genes (*p* < 0.001). In addition, a notable proportion of clock CpGs associated with at least 10 genes (26–33% of each clock), and a small proportion even with 100 or more genes (1–13% of each clock). Across all clocks, 578 CpGs associated with the expression of 1640 genes *in trans*. We hypothesized that the *trans*-genes that are informative on the mechanisms driving changes in DNAm would be associated with multiple CpGs and vice versa. Therefore, we focused our further analysis on a core set of 216 *trans*-genes that were associated with at least 5% of any of the 4 investigated clocks’ CpGs (in other words, genes which associated with either 4 Hannum Bld CpGs, 18 Horvath MT CpGs, 20 Horvath Skn/Bld CpGs, or 26 Zhang Bld/Slv CpGs), and a core set of 365 CpGs that associated with at least 10 *trans*-genes. In contrast to the *cis*-genes that were clock-specific, the *trans*-genes identified were highly concordant between clocks (Fig. 2D). For instance, all 117 genes which associated with Zhang Bld/Slv also associated with Horvath Skn/Bld. In accordance to the large overlap, each clock’s *trans*-genes showed enrichments for highly similar GO terms, with the majority of enriched terms being related to T cell activity, implying a role for T cell subtypes in progression of the epigenetic clocks (Fig. 2E; Additional file 3: Table S2).

**Fig. 3:**
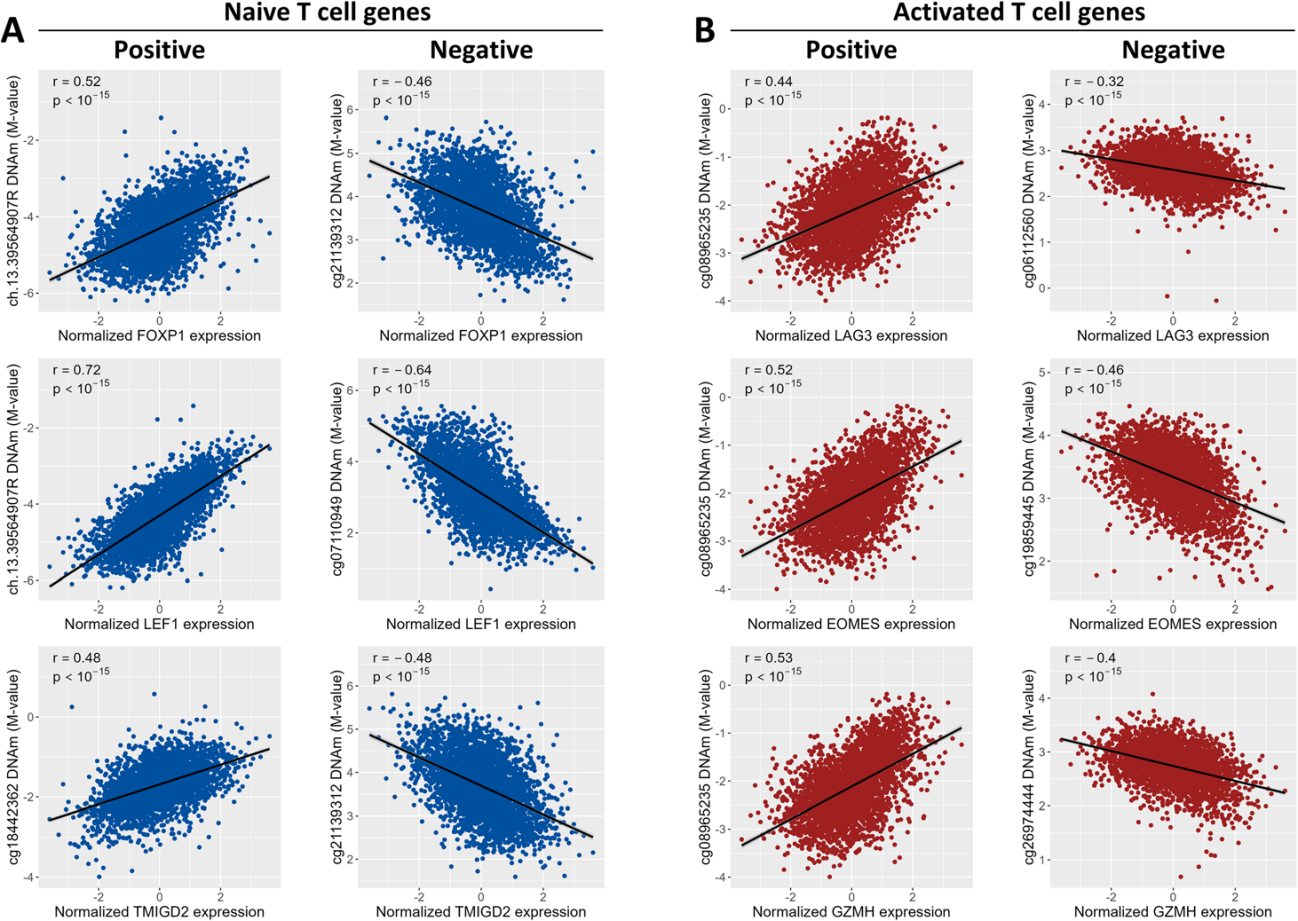
Examples of positive and negative correlations between clock CpG DNAm and gene expression (rank-inverse normal transformed), as identified in the linear regression analysis. The subfigures show the Pearson correlation (*r*) between gene expression and DNA methylation for a gene-CpG pair, as well as the *p* value for the correlation. **A** Positive and negative correlations identified for three genes with known expression in naive T-cells: *FOXP1*, *LEF1*, and *TMIGD2*. **B** Positive and negative correlations identified for three genes with known expression in activated T cells: *LAG3*, *EOMES*, and *GZMH*

A role of T cell subtypes was further indicated by clustering of the *trans*-associations. The 365 clock CpGs and the 216 *trans*-genes formed two distinct clusters, which were independent of the epigenetic clock the CpGs belonged to (Additional file 1: Fig. S2). The first cluster consisted of 152 CpGs and 93 *trans*-genes (median correlations of 0.93 and 0.95 for CpGs and genes, respectively). The second cluster consisted of 213 CpGs and 123 genes (median correlations of 0.95 and 0.94 for CpGs and genes, respectively). The genes and CpGs in the two clusters were strongly negatively correlated (median correlation of −0.93 and −0.89 for CpGs and genes, respectively), suggesting that the two clusters have opposite biological roles. Indeed, the first cluster contained genes involved in naive T cell function, such as *FOXP1* (a regulator of quiescence in T cells [13]), *LEF1* (transcription factor involved in naive T cell homeostasis [14]), and *TMIGD2* (an immune checkpoint molecule predominantly expressed by naive T cells [15, 16]). In contrast, the second cluster included genes characteristic for activated T cells, for example *LAG3* (an immune checkpoint molecule expressed by activated T cells and a subset of NK cells [17, 18]) and *EOMES* (transcription factor involved in the differentiation of effector T cells [19]), and several genes involved in degranulation (perforin and granzymes A, H, and K [20]). The correlation of these *trans*-genes with clock CpG methylation was considerable, varying between 0.44 and 0.72 for positive correlations and between −0.32 and −0.64 for negative correlations (Fig. 3). These results give an indication that the first cluster of *trans*-genes is representative of naive T cells, while the second cluster represents activated T cells.

Since aging is accompanied by a decrease in naive immune cell abundance and an increase in activated immune cells [21], we expected the expression of *trans*-genes to correlate with age. To test this, we compared our *trans*-genes with a list of age-related genes published by Peters et al. [22]. In the analysis of Peters et al., 1497 out of the 10342 investigated genes were significantly correlated with age. In our analysis, 216 out of the 14370 investigated genes were identified as *trans*-genes. Our *trans*-genes were strongly enriched for age correlation as annotated by Peters et al. (108 overlapping genes, OR = 10.0, *p* < 10^-15^). Moreover, the *trans*-genes in the first cluster (containing genes associated with naive T cells) were mostly negatively correlated with age, while the genes in the second cluster (containing genes associated with activated T cells) were usually positively correlated with age (Additional file 1: Fig. S2). Thus, the correlations of *trans*-genes with age support the notion that their expression is a signature of age-related T cell proportions.

To rule out that the observed *trans*-associations were caused by genetic variants, we compared two recently published quantitative trait locus (QTL) resources, the first containing methylation-QTLs (meQTLs) [23], and the second containing expression-QTLs (eQTLs) [24]. We reasoned that a *trans*-association driven by a genetic variant would require the variant in question to be simultaneously a *cis*-meQTL for the CpG and a *trans*-eQTL for the gene or vice versa. We tested, for each clock CpG identified in the regression analysis, whether it had a known *cis*-meQTL. Then, we tested whether the identified *cis*-meQTLs (if any) were also identified as *trans*-eQTL for the genes that significantly associated with that CpG. Only 9 out of 365 CpGs shared one or more SNP with a total of 31 *trans*-genes (Additional file 4: Table S3); the 31 *trans*-genes were also associated with 339 other CpGs for which no QTL effect was found. We also performed this analysis in the opposite direction (testing for each *trans*-gene if it is driven by *cis*-eQTLs which are also *trans*-meQTLs for any of the CpGs that significantly associated with the gene) and found that only 2 out of the 216 *trans*-genes shared a SNP with 2 clock CpGs. We conclude that genetic effects cannot explain our observations.

### Clock CpGs and *trans*-genes correlate with naive and activated T cell and NK cell phenotypes

**Fig. 4:**
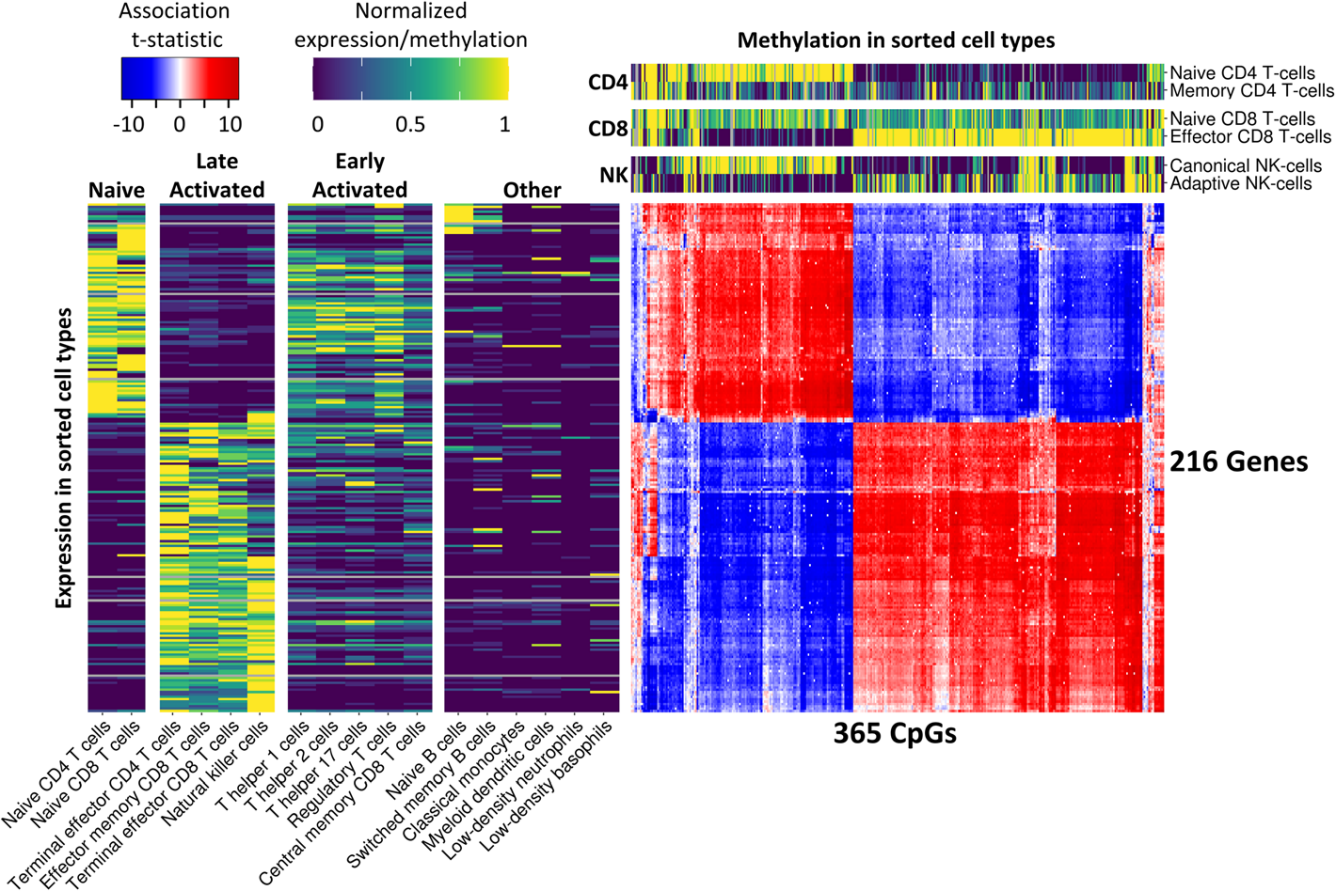
DNAm of clock CpGs and expression of *trans*-genes across sorted blood cell types. Per gene-CpG pair, the t-statistic of their association is shown in the blue-red heatmap (red = positive association, blue = negative association, white = no significant association). Only CpGs which associated with at least 10 genes and genes which associated with at least 5% of any clock were included. Genes and CpGs were clustered based on Euclidean distance. Two sidebars (viridis color scale) were included with external data, with the left sidebar depicting expression of *trans*-genes in 17 sorted blood cell types published by Monaco et al. [25], and the top sidebar depicting DNAm of clock CpGs in sorted T cell and NK cell subtypes combined from 4 datasets [13, 26–28]. For visualization, external gene expression and DNAm data were normalized so that each gene/CpG had a range between 0 and 1 across all measured cell types

To gain further insight in the involvement of specific cell types in the two clusters of clock-CpGs and *trans*-genes, we used an external dataset containing transcriptomic (RNA-seq) data from sorted blood cell types [25]. Unbiased hierarchical clustering based on *trans*-gene expression divided the cell types into four clusters (Additional file 1: Fig. S3), which we labeled based on their distinct cellular characteristics: *Naive* (containing naive T cells), *Late Activated* (containing terminal effector T cells, effector memory T cells, and NK cells), *Early Activated* (containing helper, regulatory, and central memory T cells), and *Other* (containing B cells and cells of myeloid origins; Fig. 4). The first cluster of 93 *trans*-genes was significantly higher expressed in naive cells compared to late activated cells (*p* < 10^-15^), while the opposite was the case for the second cluster of 123 *trans*-genes (*p* < 10^-15^). The cell types in the early activated cluster showed intermediate expression of both *trans*-gene clusters, while the other group showed very low expression for both clusters. These results indicate that the two *trans*-genes clusters reflect opposite gene expression patterns in naive and late activated immune cells.

Next, we performed a similar analysis using publicly available DNAm data from sorted blood cell types [13, 26–28]. In line with cell type-specific gene expression, naive and activated immune cell types from the same donor showed marked differences in DNAm of clock CpGs (Fig. 4). The 152 clock CpGs in the first cluster were consistently higher methylated in naive cells as compared with their activated counterparts (CD4 naive versus memory: *p* < 10^-15^, CD8 naive versus effector: *p* < 10^-15^, NK canonical versus adaptive: *p* < 10^-15^). The opposite pattern was found for 213 CpGs in the second cluster, which showed consistently higher DNAm in the activated cell types compared to their naive counterparts (CD4 memory versus naive: *p* < 10^-12^, CD8 effector versus naive: *p* < 10^-15^, NK adaptive versus canonical: *p* < 10^-11^). Together, these results indicate that the differences in DNAm levels between the two clusters of clock CpGs can be attributed to their differential DNAm in naive and activated T and NK cell subtypes.

Our results indicate that changes in T and NK cell subtypes are involved in progression of epigenetic clocks. However, the associations between clock CpG DNAm and gene expression (Fig. 4) were adjusted for broad white blood cell type proportions (lymphocytes, monocytes, eosinophils, basophils, and neutrophils). Therefore, it is possible that the effects of these broad blood cell types were missed. We analyzed the *trans*-associations again without correcting for white blood cell counts (all other covariates were kept the same). In this analysis, we observed 155 clock CpGs that were associated with 1044 trans-genes. This is a lower number than in our original analysis (where we found 578 CpGs associating with the expression of 1640 genes), presumably due to a combination of factors including a larger inflation of test statistics (mean inflation = 3.3, compared to only 1.4 for the original model). When filtering for genes associating with at least 5% of CpGs of any clock *in trans*, only 3 of the 216 genes remained (*TPRG1*, *EOMES*, and *KLRG1*). We therefore chose to lower the thresholds for visualization to include genes associating with at least 2.5% of any clock and CpGs associating with at least 5 genes *in trans*, resulting in 58 genes and 100 CpGs (Additional file 1: Fig. S4). All 58 *trans*-genes, as well as 83 of the 100 CpGs, were also found in the original *trans*-analysis correcting for broad cell counts (Fig. 4). Moreover, the genes and CpGs showed similar expression and DNAm patterns across cell types (Additional file 1: Fig. S4, left and top sidebars), with two distinct clusters representing naive and activated immune phenotypes. Thus, this analysis also indicated T and NK cell subsets as a main contributor to the DNAm levels of clock CpGs.

Additionally, to check the validity of our method of predicting main blood cell counts, we repeated the analysis with only the 2120 samples for which cell counts were measured, now using measured cell counts as covariates. The results from this analysis were very similar to the original analysis using predicted cell counts (Fig. 4). Adopting the same cutoffs as for the original analysis (genes which associate with at least 5% of any clock and CpGs associating with 10 genes), we identified 263 clock CpGs associating with 154 *trans*-genes (Additional file 1: Fig. S5). This a lower number than the original analysis, presumably due to the lower sample size, but the sets were similar nonetheless: 252 out of 263 CpGs and 148 out of the 154 genes were also identified in the original analysis with the complete set of 3132 samples. Also, the genes and CpGs formed the same two clusters representing naive and activated immune phenotypes (Additional file 1: Fig. S5). Therefore, the prediction of main cell counts in order to analyze the complete set of samples did not affect our findings.

### Age prediction by epigenetic clocks is influenced by blood cell types

**Fig. 5:**
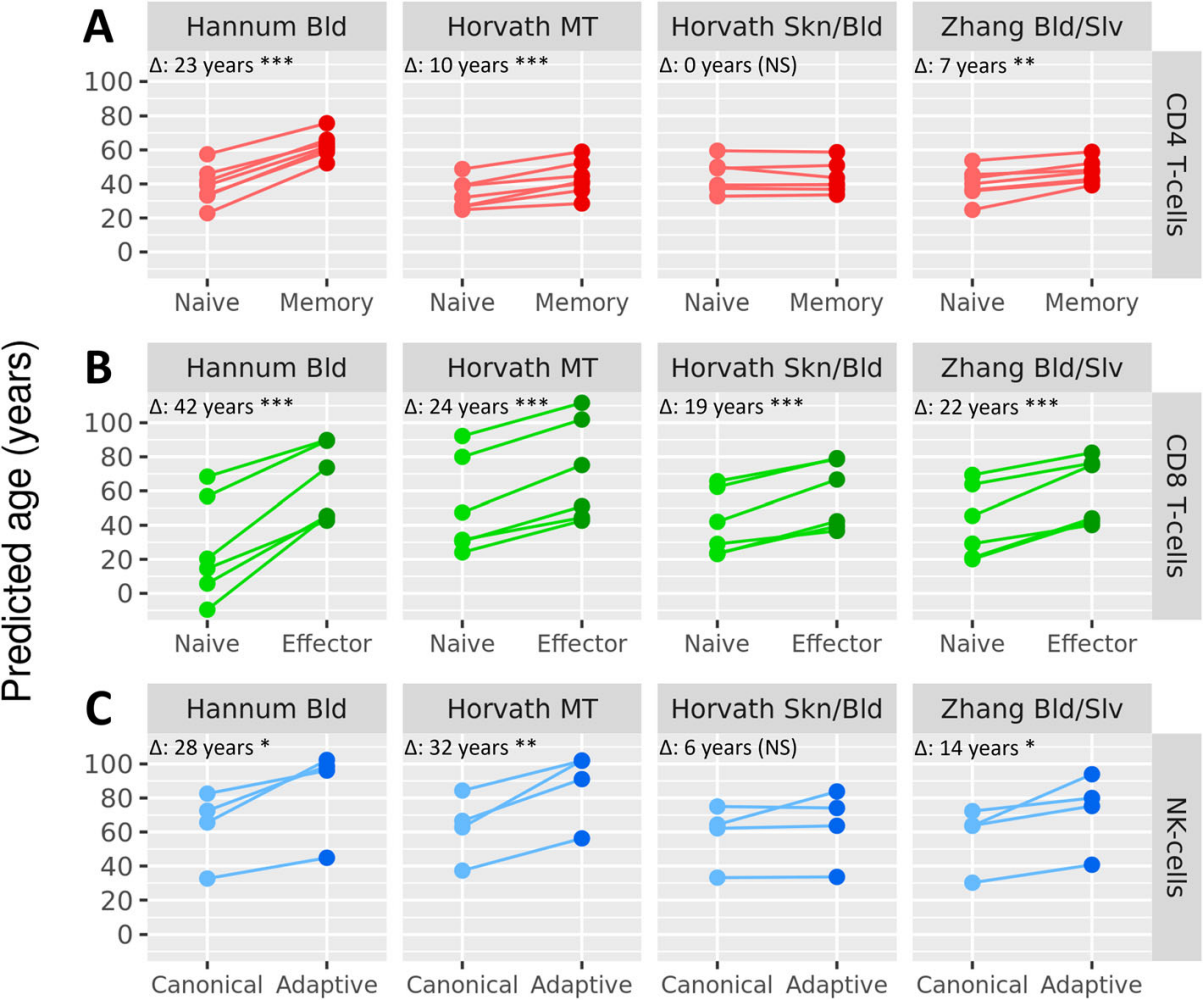
Age prediction by epigenetic clocks in sorted blood cell types. Age was predicted by applying the 4 investigated epigenetic clocks to DNAm data from 4 publicly available datasets generated in sorted blood cells [13, 26–28]. For each subfigure, the median difference in age prediction of each clock between the naive and activated phenotype is shown in the top left of each panel (Δ: *x* years), and significance is marked by stars (*p < 0.05, **p < 0.01, NS not significant). Naive and activated phenotypes were obtained from the same donors in all instances, and samples originating from the same donor are connected with a line. **A** The data published by Garaud et al. [13] and Pitaksalee et al. [26] were generated in sorted CD4^+^ naive and memory T cells (*n* = 7). **B** The data published by Schlums et al. [27] and Rodriguez et al. [28] were generated in sorted CD8^+^ naive and effector T cells (*n* = 6). **C** The data published by Schlums et al. [27] also contained canonical and adaptive NK cell samples (*n* = 4)

Finally, we tested whether the differential DNAm of clock CpGs in naive and activated cell subtypes influenced the age prediction of epigenetic clocks. To this end, we applied the 4 clocks to the different blood cell subtypes from the external datasets. Overall, the 4 clocks predicted naive CD4^+^ T cells, CD8^+^ T cells, and canonical NK cells to be younger than their activated counterparts from the same donor (*p* < 0.05; Fig. 5) with the Horvath Skn/Bld clock as a single exception for CD4^+^ T cells and NK cells. For CD4^+^ T cells, Hannum Bld, Horvath MT, and Zhang Bld/Slv predicted memory CD4^+^ T cells 7 to 23 years older than naive CD4^+^ T cells from the same donor (Fig. 5A). For CD8^+^ T cells, all clocks predicted effector cells to be older than naive cells from the same donor with differences ranging from 19 to 42 years (Fig. 5B). Lastly, adaptive NK cells were predicted to be 14 to 32 years older by Hannum Bld, Horvath MT, and Zhang Bld/Slv compared to canonical NK cells from the same donor (Fig. 5C). The largest difference in age prediction was consistently found for the Hannum Bld and Horvath MT clocks. Together, these results show that age prediction by epigenetic clocks strongly differs between immune cell subtypes.

## Discussion

Our analysis revealed that all four investigated epigenetic clocks were able to accurately predict age in whole blood and were highly concordant in terms of age prediction in line with the overlap in or correlation between CpGs of the clocks. Strikingly, we found that DNAm of clock CpGs was frequently associated with gene expression *in trans* and involved similar genes for each clock with a role in T cell function. Further analysis of public DNAm and gene expression data of purified immune cells highlighted that both clock CpG DNAm and their *trans*-associated genes distinguished naive and activated T and NK cells. Finally, the clocks predicted activated cells to be up to decades older than naive cells despite being from the same donor.

Interestingly, our findings were congruent for all investigated clocks, despite the different tissues and data sets on which they were trained. This was especially remarkable for the Horvath MT clock, which was trained on samples from 27 different tissues, but still gave overall similar results to the three other clocks, which were trained exclusively on whole blood samples (Hannum Bld) or mostly on whole blood plus buccal cells and fibroblasts (Horvath Skn/Bld) or saliva samples (Zhang Bld/Slv). A possible explanation for this finding is that a large percentage (37%) of Horvath-MT’s training set consisted of whole blood samples, which makes it plausible that some CpGs in this clock are predictive of blood cell proportions. A plausible alternative explanation is that the prediction by epigenetic clocks in non-blood tissues is affected by tissue-resident T cells and NK cells, which are present in many tissues included in Horvath MT’s training set [29–33]. The latter interpretation would also explain why epigenetic clocks trained primarily or even exclusively on whole blood samples are relatively accurate when applied to other tissues [3–5, 34].

It should be noted that our aim was not to find or imply any direct causal link between clock CpG DNAm and *trans*-gene expression. Instead, we characterized clock CpGs by analyzing their correlation with *trans*-gene expression. The inspection of the function and cell-specific expression of these *trans*-genes subsequently led to the interpretation that the *trans*-genes and the correlating clock CpGs were both markers of the same blood cell type proportions, namely naive and activated T and NK cells. Hence, our data support the interpretation that age-related changes in clock CpG methylation and *trans*-gene expression have shifts in blood cell types as a common cause, which leads to a notable correlation between the two.

Our analyses can explain why the investigated clocks can accurately predict age, namely by detecting the ratio between naive and activated T and NK cells in a sample. The reduction of naive cells and the accumulation of activated immune cells is a well-established feature of increasing age and immuno-senescence [21]. However, a limitation of our analysis is that it could not determine which percentage of the clocks’ predictive ability can be attributed to T and NK cells. For example, other cell type proportions may also play a role, but our design is not optimal to detect the influence of broad cell types like granulocytes, lymphocytes, and monocytes. Nevertheless, when we repeated the analysis of the associations of clock-CpGs with gene expression in *trans* without correcting for these broad cell types, we again highlighted the involvement of T cells. As expected, this analysis suffers from high inflation of test statistics. Although we corrected for this inflation using a method that is designed to be less sensitive to true-positive associations [35], the analysis may not provide a complete picture of *trans*-genes expressed in broad cell types. DNAm profiling of purified cell types with accurate data on their relative proportions from larger series of donors is required to definitively answer this question.

**Fig. 6:**
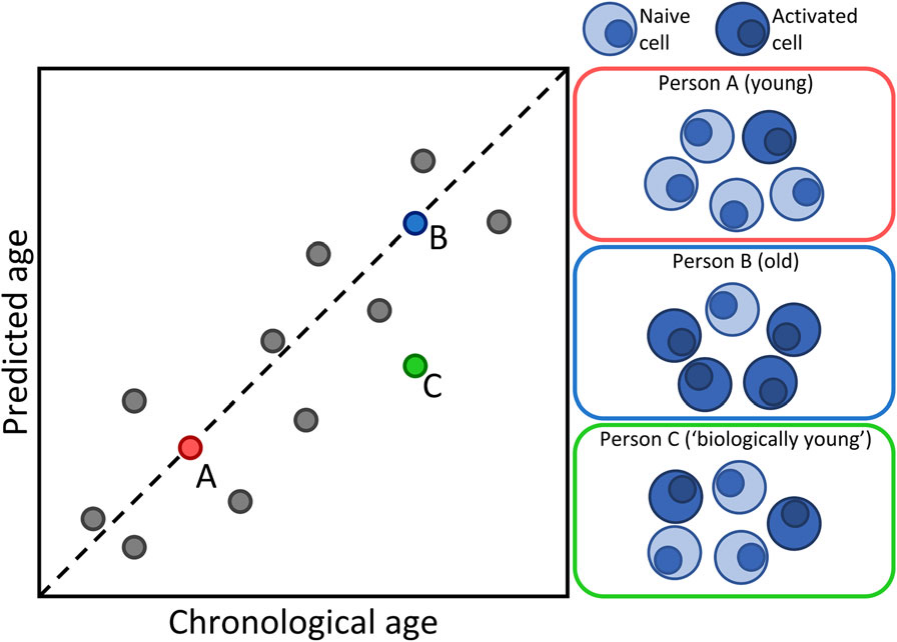
Proposed model for the influence of blood cell proportions on age prediction by epigenetic clocks. Three hypothetical people are highlighted. Person A is young and has a blood cell composition consisting of mostly naive immune cells, leading to epigenetic clocks rightfully predicting them to be young. Conversely, Person B is old and has a relatively high proportion of activated immune cells in their blood, causing the epigenetic clocks to estimate them to be old. For Person C, who has the same chronological age as Person B but a blood cell composition closer resembling that of a young person, epigenetic clocks would likely underestimate their age. Note that our data indicate that the prediction errors of the various clocks (e.g., due to measurement error of the arrays) also contribute to deviations between chronological and predicted age and hence biological phenomena do not explain deviations in full

On the basis of our findings, we propose a model where the age predictions of epigenetic clocks in blood depend on proportions of naive and activated immune cell types in a sample (Fig. 6). According to this model, the relative proportion of activated T and NK cells increases with age at the expense of naive cells, which, due to their distinct DNAm at clock CpGs, is expressed as the “ticking” of epigenetic clocks. The model also implies that deviation of the predicted age from chronological age may stem from a naive-activated cell ratio that is uncharacteristic for a certain chronological age. Therefore, our results may contribute to the interpretation of studies in which such deviations are considered as markers of biological age and associated with disease outcomes. For example, many age-related health outcomes are accompanied by a pro-inflammatory state, which skews immune cell proportions towards activated phenotypes. Examples of such health outcomes are Alzheimer’s disease [36], cardiovascular disease [37], and cytomegalovirus infection [38]. This presents a potential explanation for the observed correlation of Horvath MT’s age prediction with these outcomes [39–41]. Finally, our results may guide the selection of age-related phenotypes to study with epigenetic clocks depending on prior knowledge of the involvement of T and NK cells. An important consideration when interpreting deviations of predicted age from chronological age is that these deviations also reflect measurement errors in the methylation arrays and prediction errors of the clocks. Measurement errors have been reported previously [42], and although we observed that deviations were highly correlated between the various clocks, substantial differences remained and extreme deviations were not reproduced across clocks. Hence, deviations do not have a fully biological interpretation.

## Conclusions

The ability of epigenetic clocks to predict chronological age involves their ability to detect changes in proportions of naive and activated immune blood cells. This finding may contribute to the interpretation of associations between clock-derived measures and age-related health outcomes.

## Methods

### Cohorts

This study was performed using DNAm and RNA-seq data generated within the Biobank-based Integrative Omics Studies Consortium (BIOS Consortium). All data were generated in whole blood samples originating from 6 Dutch biobanks: Cohort on Diabetes and Atherosclerosis Maastricht (CODAM) [43], LifeLines (LL) [44], Leiden Longevity Study (LLS) [45], Netherlands Twin Register (NTR) [46], Rotterdam Study (RS) [47], and the Prospective ALS Study Netherlands (PAN) [48]. NTR is a biobank of twins, so a random person was selected from each twin pair to ensure that samples were unrelated. We selected samples from all cohorts for which DNAm data, RNA-seq data, and genotype data were all available and for which all of the data passed quality control (QC), which was the case for 3132 samples. To prevent sample mix-ups from influencing the results, we verified the identity of all DNAm and RNA-seq samples with genotype data using *OmicsPrint* [49].

Age, sex, and cell counts of major white blood cell types were obtained for each cohort (measured cell types: lymphocytes, monocytes, neutrophils, eosinophils, basophils). Cell counts were available for 2120 samples (69% of the total dataset). In line with previous work [50–52], a prediction model for cell counts was subsequently trained on these cell counts using *wbccpredictor*, which fits a multivariate partial-least-squares model, including age and gender, on the DNA methylation data. The prediction workflow is available at https://molepi.github.io/DNAmArray_workflow/05_Predict.html. Predicted cell counts were used for all samples.

### DNA methylation data

DNAm data were generated by bisulphite-converting 500 ng of genomic DNA from whole blood samples using the EZ DNA Methylation kit (Zymo Research, Irvine, CA, USA). Subsequently, bisulphite-converted DNA was hybridized onto Infinium HumanMethylation450 BeadChip arrays (Illumina, San Diego, CA, USA), and signal intensities were measured on an Illumina iScan BeadChip scanner according to the manufacturer’s protocol.

A detailed description and R code of the pre-processing workflow, including the quality control and normalization, is described in DNAmArray, which is publicly available at https://molepi.github.io/DNAmArray_workflow/. In brief, IDAT files were read into R using *minfi* [53], after which sample-level QC was performed with *MethylAid* [54]. Low-quality samples were defined based on 4 QC-measures using information provided by the control probes on the array and 1 measure was based on call rate (>95%) as shown in Additional file 1: Fig. S6, which led to the exclusion of 168 samples. Probe-level QC was based on detection *p* value (*p* < 0.01), number of beads available (≤ 2) or zero values for signal intensity. Resulting probes with more than 5% missing values were removed. Normalization was done using functional normalization as implemented in *minfi*, using five principal components extracted using the control probes for normalization.

Sugden et al. analyzed the reliability of measurements of DNAm from Illumina 450K arrays using repeated measurements [42]. First, we downloaded the reported reliability of 450K CpGs as expressed using the intraclass correlation (ICC) and selected the ICC for all CpGs incorporated in the 4 clocks we investigated. The median ICC of clock CpGs was 0.36 (Additional file 1: Table S4). For context, ICCs below 0.4 were considered “poor,” those between 0.4 and 0.6 were considered “fair,” between 0.6 and 0.75 “good,” and above 0.75 “excellent.” Next, we also calculated the ICCs for clock CpGs using our own data on 15 samples for which we had a technical replicate (30 samples in total; ICC was calculated for each CpG using a mean-rating (*k* = 2), absolute-agreement, 2-way random-effects model). We found that the reliability of clock CpGs was markedly higher in our data as compared with the analysis of the replicates available to Sugden et al. with median ICC of 0.68, and reaching good or even excellent reliability for most CpGs (Additional file 1: Table S4). We concluded that the DNAm data we used in our analysis compare favorably to data used in other reports and can be considered sufficiently reliable.

### Gene expression data

A detailed description of the generation and processing of the RNA sequencing (RNA-seq) data can be found in previously published work of our group [55]. In short, globin transcripts were removed from whole blood RNA using the Ambion GLOBINclear kit and subsequently processed for RNA-sequencing using the Illumina TruSeq version 2 library preparation kit. RNA libraries were paired-end sequenced using Illumina’s HiSeq 2000 platform with a read length of 2 × 50 bp, pooling 10 samples per lane. Reads which passed the chastity filter were extracted with CASAVA. Quality control was done in three steps: initial QC was performed using FastQC (v0.10.1), adaptor sequences were removed using *cutadapt*, and read ends with insufficient quality were removed with *Sickle*. Reads were aligned to the human genome (hg19) using *STAR* (v2.3.0e). To avoid reference mapping bias, all GoNL SNPs (http://www.nlgenome.nl/?page_id=9) with MAF > 0.01 in the reference genome were masked with N. Read pairs with at most 8 mismatches, mapping to at most 5 positions, were used. Gene counts were calculated by summing the total number of reads aligning to a gene’s exons according to Ensembl, version 71. Samples for which less than 70% of all reads mapped to exons were removed, as shown in Additional file 1: Fig. S7; this was the case for 11 samples. Summary statistics of the RNA-seq samples which passed QC are shown in Additional file 1: Table S5.

For analysis, genes were filtered to include only protein-coding genes (as annotated by Ensembl, version 71) with sufficient expression in our dataset (median count >1), which resulted in the inclusion of 14370 genes. Raw counts of these genes were subsequently transformed into log counts per million (CPM) values. Additionally, to ensure that the RNA-seq data were normally distributed, a rank-inverse normal (RIN) transformation was performed per gene.

### Epigenetic clocks

Four epigenetic clocks were studied: the blood clock developed by Hannum et al. (Hannum Bld) [2], the multi-tissue clock developed by Horvath (Horvath MT) [3], the skin/blood clock developed by Horvath et al. (Horvath Skn/Bld) [4], and the blood/saliva clock developed by Zhang et al. (Zhang Bld/Slv) [5]. These clocks are described in Table 1. Together, the four epigenetic clocks comprised 1147 unique CpGs. One CpG from Horvath Skn/Bld (cg14614643) did not pass QC in our DNAm data and was therefore excluded from our analyses (i.e., 1146 CpGs were included). The four epigenetic clocks were used to predict chronological age in all 3132 samples for which methylome data were available. To this end, the coefficients of all clock CpGs were downloaded (available in their respective publications [2–5]). Beta-values of the clock CpGs were used as input for all clocks. For Horvath MT and Horvath Skn/Bld, predicted ages were transformed according to the authors’ instructions [3, 4]. For Zhang Bld/Slv, DNAm values were normalized according to the authors’ instructions, so that all samples had a mean of 0 and a standard deviation of 1 across all 450K CpGs [5].

### Correlation between clock CpGs

To obtain an impression of the degree to which the CpGs comprising the 4 clocks capture the same information, we analyzed the correlation of their methylation values. The clocks were trained using elastic net, a penalized regression method which aims to limit internal correlations within a predictor. Therefore, it is expected that most correlations in DNA methylation of CpGs within and between clocks are (very) low. Therefore, we reported the percentage of each clock’s CpGs which had a correlation with another CpG’s methylation level of at least 0.5, 0.7, and 0.9 within the same clock and in the 3 other clocks. For correlations between clocks, instances where the same CpG was included in two clocks were included as a correlation of 1; for correlations within a clock, the correlation of the CpGs with itself was excluded.

### Enrichment for genomic features

For annotation of CpG islands (CGIs), the CGI-track from UCSC was queried (genome hg19) using *rtracklayer*. CpGs from the 450K array were annotated according to 3 CGI-centric features: CGIs (as annotated by UCSC), shores (2 kb regions flanking the CGIs), and non-CGI.

Chromatin immunoprecipitation sequencing (ChIP-seq) data of 6 histone modifications (H3K4me1, H3K4me3, H3K27ac, H3K36me3, H3K9me3, and H3K27me3) in PBMCs were downloaded from the Epigenomics Roadmap project [12] using *AnnotationHub* [56]. For histone modifications with relatively broad enrichment regions (H3K4me1, H3K4me3, and H3K27ac), we used the output of the *broadPeak* peak calling method, while for histone modifications with relatively narrow enrichment regions (H3K36me3, H3K27me3, and H3K9me3), we used the output of the *narrowPeak* peak calling method. Peak calling methods are described in detail by Kellis et al. [57]. Additionally, chromatin state segments, imputed from 5 core histone modifications (H3K4me1, H3K4me3, H3K36me3, H3K9me3, H3K27me3) using *ChromHmm*, were downloaded from this resource [12] using *AnnotationHub* [56]. Since our DNAm and RNA-seq data were generated in whole blood samples, we chose PBMCs as a reference epigenome (epigenome E062 according to the Epigenomics Roadmap nomenclature).

For enrichment analysis of the annotations described above, we calculated odds ratios (OR) of clock CpGs being annotated with a genomic feature compared to all non-clock CpGs from the 450K array being annotated as such. Statistical significances were obtained using Fisher’s exact test. Multiple testing correction was done using the Bonferroni method with 9 independent variables (3 CGI-centric features and 6 histone modifications), since the 15 chromatin states were derived from the 6 histone modifications.

### Associations between DNA methylation and gene expression

To analyze the correlation between DNAm of clock CpGs and gene expression, a linear regression analysis was performed for each CpG. Only autosomal genes and CpGs were considered, which led to the exclusion of a CpG from Horvath Skn/Bld which mapped to the X-chromosome (cg01892695). The regression was performed using the R package *cate*, which implements a method to run a linear regression model including both known covariates and estimated latent factors in the data causing residual confounding [58]. Only samples for which both DNAm data and RNA-seq data were available and for which none of the model covariates were missing were included; this resulted in a total of 3132 samples. The full regression model is shown below:

$$ {\mathrm{Expression}}_{\mathrm{gene}\ \mathrm{x}}={\mathrm{DNAm}}_{\mathrm{CpG}\ \mathrm{y}}+\mathrm{Biobank}+\mathrm{Age}+\mathrm{Sex}+\mathrm{Basophil}\%+\mathrm{Eosinophil}\%+\mathrm{Lymphocyte}\%+\mathrm{Monocyte}\%+\mathrm{Bisulphite}\ \mathrm{Plate}+\mathrm{Sentrix}\ \mathrm{Position}+\mathrm{Flowcell}\ \mathrm{Number}+\mathrm{Latent}\ \mathrm{Factor}\ 1\dots 5+\upvarepsilon $$

where *Biobank* refers to the 6 cohorts comprising the data, and *basophil %*, *eosinophil %*, *lymphocyte %*, and *monocyte %* are the percentages of basophils, eosinophils, lymphocytes, and monocytes. Neutrophil percentages were excluded from the model to prevent collinearity given the extremely high correlation with lymphocyte percentage (*r* = −0.96). Finally, *Bisulphite Plate* is the plate used for bisulphite-converting DNA prior to DNAm measurement, *Sentrix Position* is the sample’s position on the 450K array, *Flowcell Number* is the HiSeq 2000 flowcell used for RNA-seq measurement, and *Latent Factor 1…5* refers to the 5 latent factors estimated by *cate*.

Any residual biases or inflations in the test-statistics generated by this model were estimated and corrected using the R package *bacon* [35]. This correction was performed separately for each CpG, correcting the *t* statistics and *p* values of its associations with all 14370 investigated genes.

From the regression analysis, we obtained a 1146 x 14370 matrix of *t* statistics and *p* values. Each entry in these matrices was indicative of the association between the DNAm of one clock CpG and the expression of one gene; examples of positive and negative associations are shown in Fig. 3. Associations *in cis* and *in trans* were analyzed separately. For associations *in cis*, only gene-CpG pairs within 100 kB of each other were analyzed. For associations *in trans*, only gene-CpG pairs which were over 5 MB away from each other or localized on different chromosomes were analyzed. After making these selections, the associations *in cis* and *in trans* were separately corrected for multiple testing using the Bonferroni method.

For the *trans*-associations, a core set of genes and CpGs was selected. We selected genes which were significantly associated (*p*_Bonf_ < 0.05) with at least 5% of the CpGs comprising any of the 4 investigated clocks (in other words, genes which associated with either 4 Hannum Bld CpGs, 18 Horvath MT CpGs, 20 Horvath Skn/Bld CpGs, or 26 Zhang Bld/Slv CpGs), and we selected CpGs which were significantly associated with at least 10 genes. The resulting set of 216 genes and 365 CpGs was visualized in a heatmap, plotting the *t* statistic as found in the regression analysis for each gene-CpG pair. Genes and CpGs were clustered using hierarchical clustering based on Euclidean distance.

To confirm the validity of our analysis, we performed a permutation test where we randomized the sample identifiers of the DNAm data 1000 times, then repeated the analysis and counted the total number of Bonferroni-significant *trans*-associations. Our actual analysis identified 50029 associations, while all 1000 permutations identified 0 associations (*p* < 0.001).

Genes associating with at least 1 CpG *in cis* and genes associating with at least 5% of any clock’s CpGs *in trans* were tested for Biological Pathway Gene Ontology enrichment using the R package *clusterProfiler* [59], with the gene background set to all 14370 genes analyzed in the TWAS. GO-term network visualization was performed using ReviGO with standard settings [11].

### External datasets on sorted blood cells

To investigate whether the levels of clock CpG DNAm and expression of associated *trans*-genes were specific for blood cell types, we downloaded publicly available transcriptomic and methylomics datasets generated in sorted blood cells. For transcriptomic data, a dataset measured in 29 sorted blood cell types from 4 donors was downloaded [25]. For clearer visualization, 17 cell types were selected for further analysis (as shown in Fig. 4). For DNAm data, a total of 4 publicly available 450K datasets were downloaded. The data published by Garaud et al. [13] and Pitaksalee et al. [26] were generated in sorted CD4^+^ naive and memory *T* cells (*n* = 7). The data published by Schlums et al. [27] and Rodriguez et al. [28] were generated in sorted CD8^+^ naive and effector *T* cells (*n* = 6). The data published by Schlums et al. [27] also contained canonical and adaptive NK cell samples (*n* = 4). For each dataset, we selected samples to ensure that a naive and activated sample would be available for each included donor, and we included only samples from healthy donors with no prior treatment. This meant we selected only resting CD4^+^ *T* cells from Garaud et al. (excluding stimulated samples, *n* = 3), CD4^+^ *T* cells from healthy controls from Pitaksalee et al. (excluding rheumatoid arthritis samples, *n* = 4), naive and effector CD8^+^ *T* cells and CD56^dim^ CD57^-^ canonical NK cells and CD56^dim^ CD57^bright^ EAT2- adaptive NK cells from Schlums et al. (excluding other NK cell subtypes from the same donors, *n* = 4), and naive and TEMRA CD8^+^ *T* cells from Rodriguez et al. (excluding effector memory *T* cells from the same donors, *n* = 2).

Levels of CpG DNAm and gene expression were normalized to have a minimum of 0 and a maximum of 1 by applying the following formula per gene/CpG:

$$ {\mathrm{X}}_{\mathrm{norm}}=\left(\mathrm{X}-\min \left(\mathrm{X}\right)\right)/\max \left(\mathrm{X}-\min \left(\mathrm{X}\right)\right) $$

where *X* is the median expression or DNAm level in one cell type, and *min(X)* and *max(X)* refer to the lowest and highest value of that gene/CpG across all cell types. Normalized expression and DNAm data from the external datasets were visualized following the clustering order of genes and CpGs in the heatmap of the *trans*-associations (gene and CpG entries which were not present in the external datasets were set to NA).

To test whether the genes/CpGs in the heatmap’s main clusters (2 clusters for both genes and CpGs) were differentially expressed/methylated across blood cell types in the external data, a series of *t* tests was performed. For the external gene expression data, the median normalized expression of each gene was calculated in naive and late activated cell types, and a *t* test was used to test for differential expression in naive cell types versus late activated cell types (this analysis was performed separately for genes in the top cluster and those in the bottom cluster). For the external DNAm data, the median normalized DNAm of each CpG was calculated in each of the 6 cell types. Differential DNAm of CpGs was separately tested for the left and right cluster using *t* tests in three settings: Naive CD4+ *T* cells versus memory CD4+ *T* cells, naive CD8+ *T* cells versus effector CD8+ *T* cells, and canonical NK cells versus adaptive NK cells.

The epigenetic clocks were applied to predict age in the external DNAm samples. Differences between naive and activated phenotypes were tested using paired *t* tests.

## Supplementary Information

**Additional file 1: Supplementary tables and figures.** Contains Fig. S1-S7 and Table S4 and S5.

**Additional file 2: Table S1.** All associations between clock CpG methylation and expression of genes in cis and in trans as identified by the regression analysis.

**Additional file 3: Table S2.** Gene ontology (GO) enrichments of the trans-genes identified in the regression analysis.

**Additional file 4: Table S3.** Overlap between meQTLs affecting clock CpG DNAm and eQTLs affecting trans-gene expression.

**Additional file 5.** Review history.

## Data Availability

The main dataset of 3132 whole blood samples is available from the European Genome-Phenome Archive (EGAC00001000277) [60]. Scripts for the analyses are available at GitHub [61] and Zenodo [62] under an open source MIT license. All external datasets used in this study are publicly available from Gene Expression Omnibus (GEO) under accession numbers GSE71825 [12], GSE107011 [24], GSE121192 [25], GSE66562 [26], and GSE83156 [27].
