# Supplementary material for: Functional genomics analysis identifies T and NK cell activation as a driver of epigenetic clock progression
Source: Genome Biol. 2022 Jan 14;23:24. doi: 10.1186/s13059-021-02585-8 (PMC8759260; doi:10.1186/s13059-021-02585-8)
Supplement: Supplementary file 1 — Additional file 1: Supplementary tables and figures. Contains Fig. S1-S7 and Table S4 and S5. [file 13059_2021_2585_MOESM1_ESM.docx]

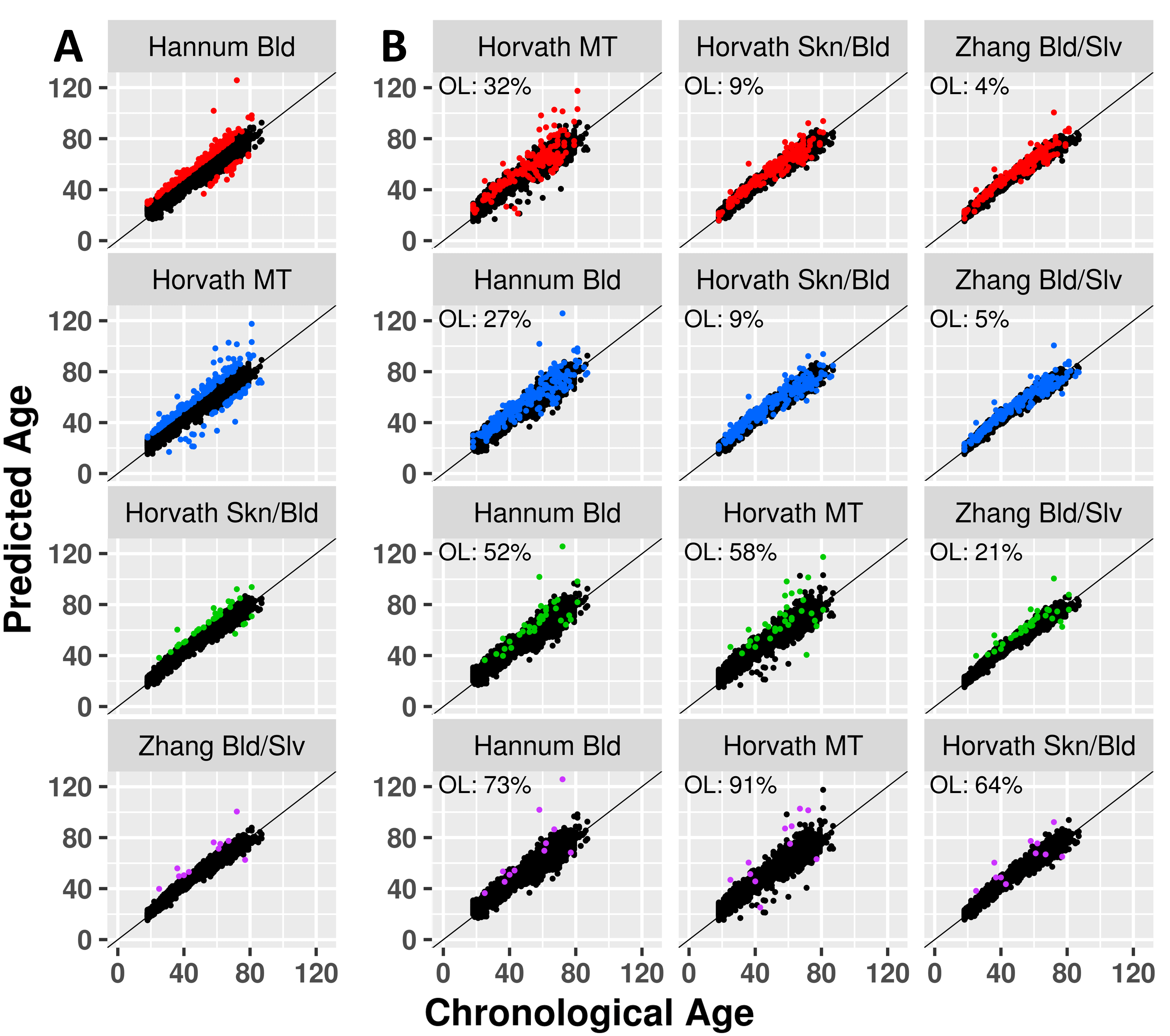


**Fig. S1.** Agreement of epigenetic clocks in extreme individuals. (**A**) For each clock, the individuals in which predicted age deviated from chronological age by at least 10 years were colored. (**B**) The predicted ages of the colored individuals per clock according to the 3 other clocks.

**Table S1.** All associations between clock CpG methylation and expression of genes *in cis* and *in trans* as identified by the regression analysis.

*Supplied separately in Additional File 2.*

**
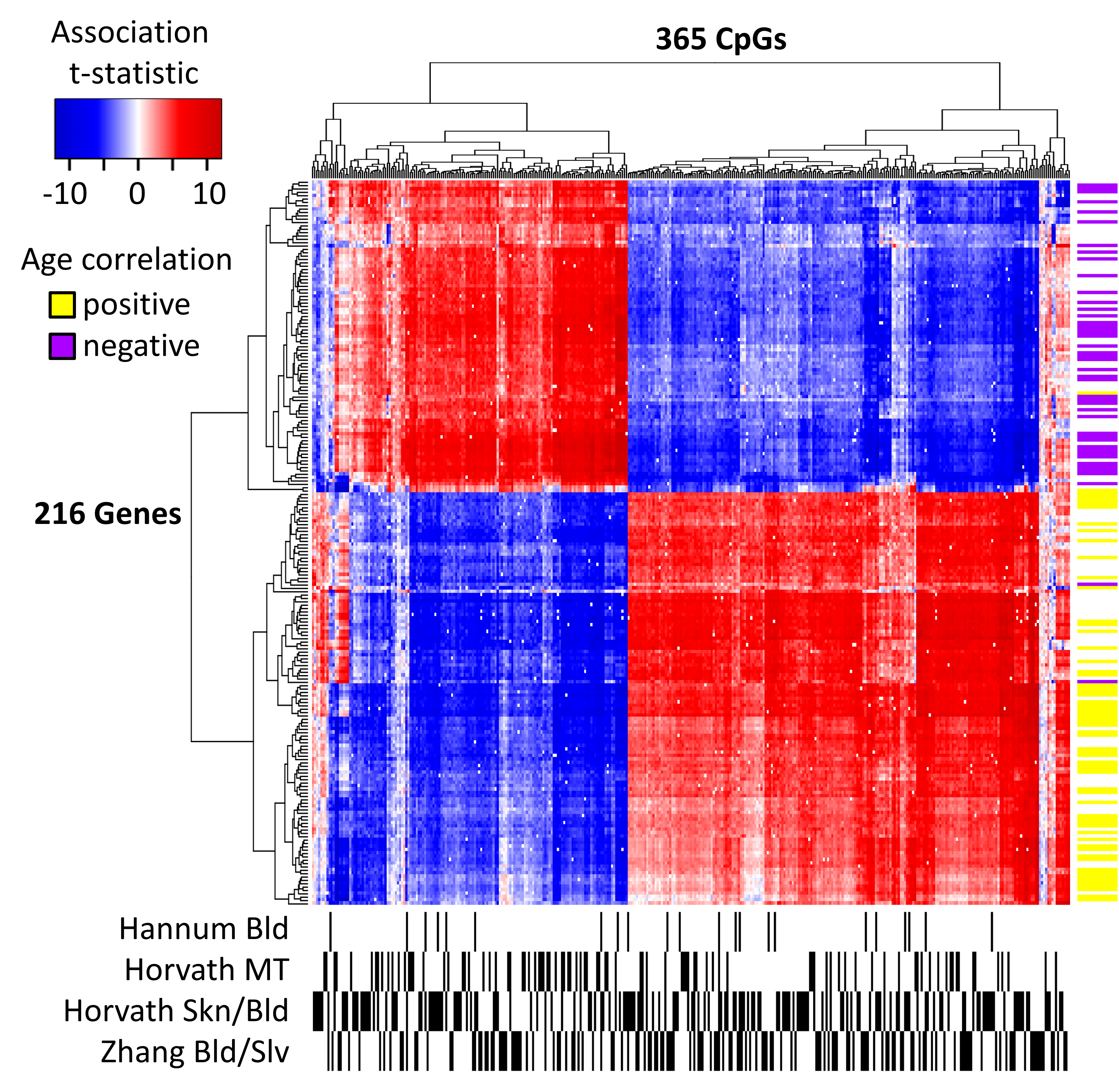
**

**Fig. S2.** Associations between DNAm of clock CpGs and expression of genes *in trans* (>5MB distance or located on different chromosomes). Per gene-CpG pair, the t-statistic of their association is shown in the blue-red heatmap (red = positive association, blue = negative association, white = no significant association). Only CpGs which associated with at least 10 genes and genes which associated with at least 5% of any clock were included. Genes and CpGs were clustered based on Euclidean distance. Column sidebar indicates whether each CpG is part of any of the 4 investigated epigenetic clocks (black = yes, white = no). Row sidebar indicates whether the each gene correlates with age according to Peters et al. [20] (yellow = positive correlation, purple = negative correlation, white = no significant correlation).

**Table S2.** Gene ontology (GO) enrichments of the *trans*-genes identified in the regression analysis.

*Supplied separately in Additional File 3.*

**Table S3.** Overlap between meQTLs affecting clock CpG DNAm and eQTLs affecting *trans*-gene expression.

*Supplied separately in Additional File 4.*


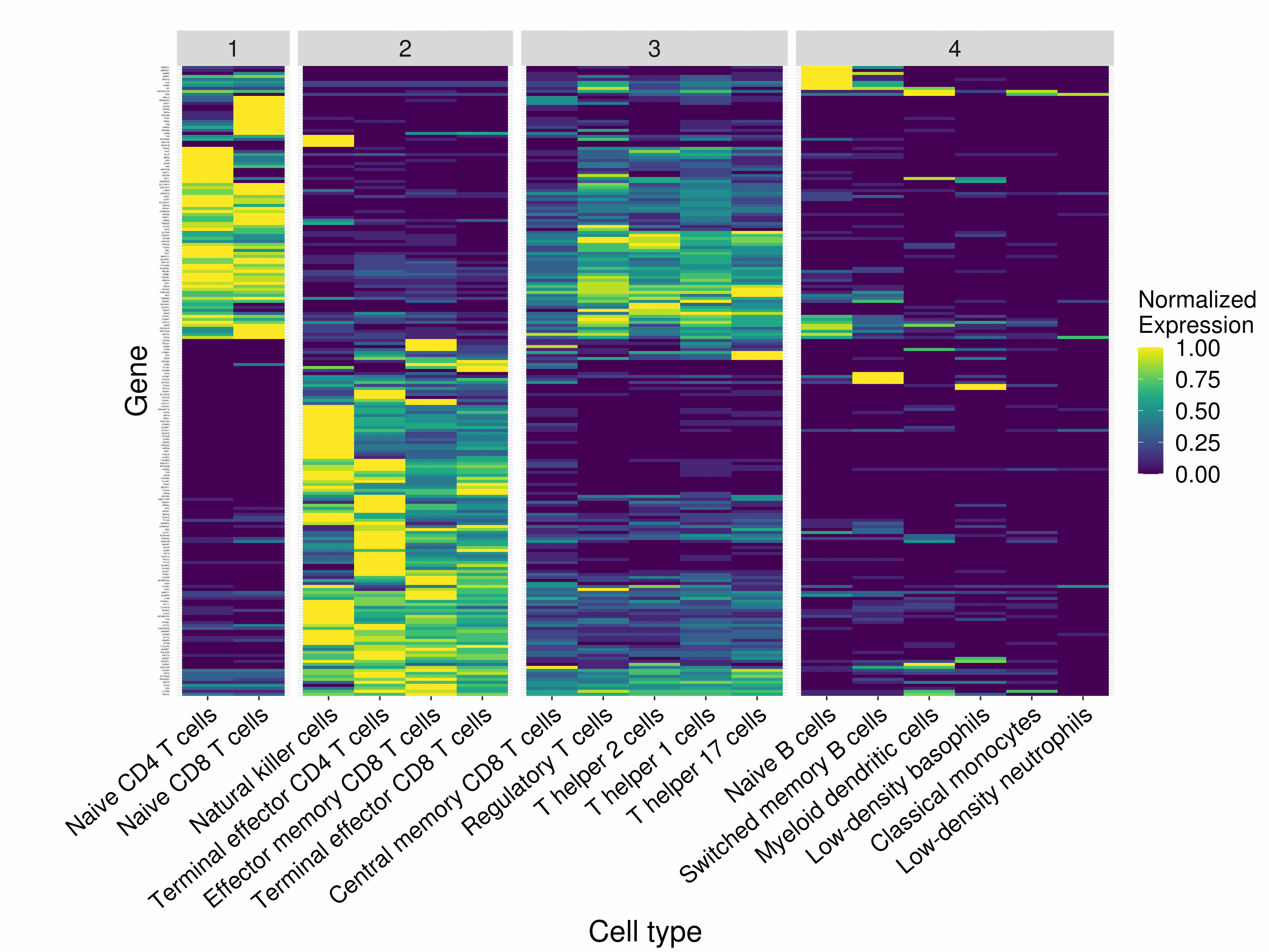


**Fig. S3.** Unbiased clustering of sorted blood cell types based on *trans*-gene expression. External gene expression data measured in 17 sorted blood cell types were obtained from Monaco et al. [21]. Expression values were normalized so that each gene had an expression range of 0-1 across all measured cell types. Genes and cell types were clustered based on Euclidean distance, and cell types were divided into 4 clusters based on expression profiles.

**
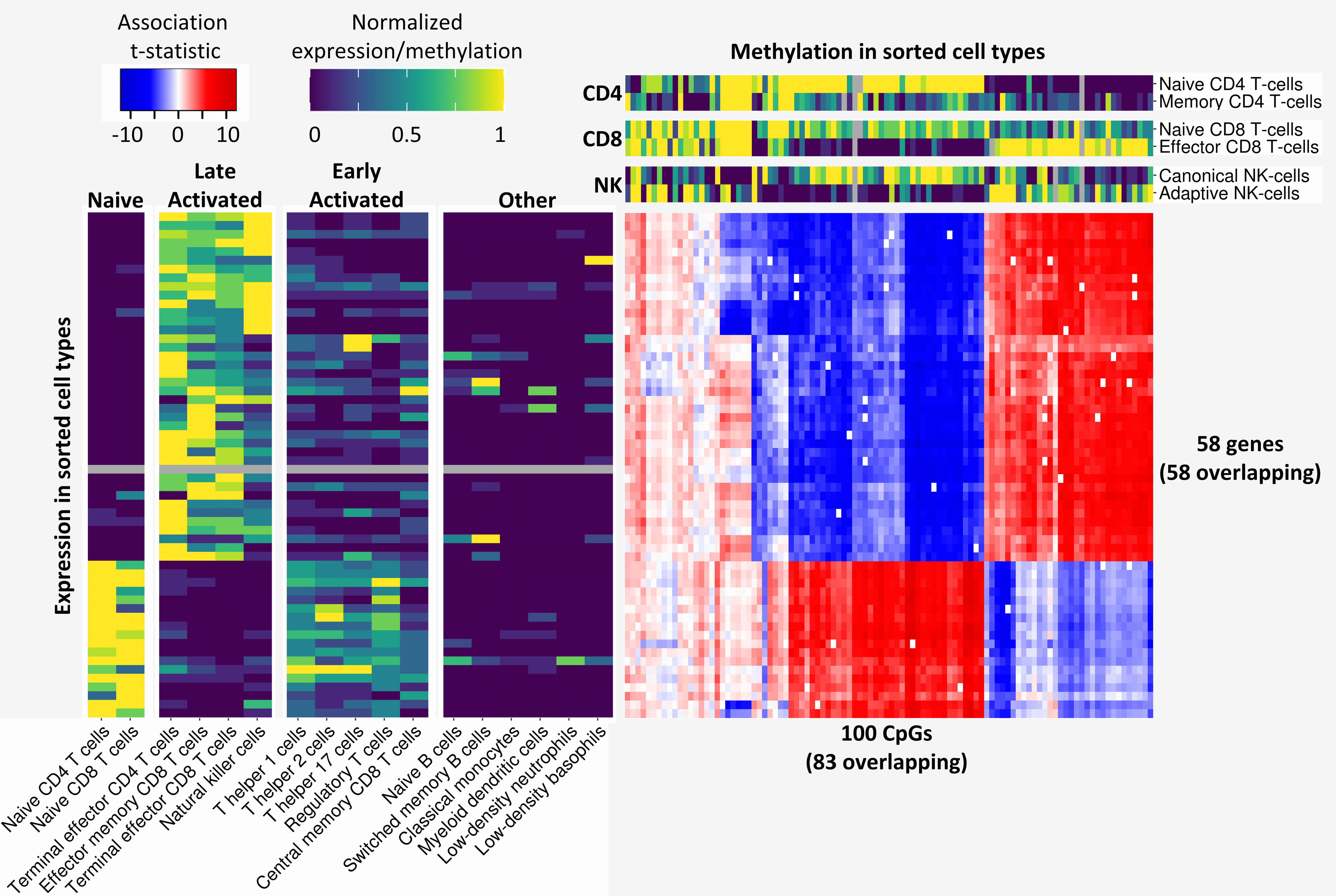
**

**Fig. S4.** Associations between DNAm of clock CpGs and expression of genes *in trans* without correction for white blood cell counts. Per gene-CpG pair, the t-statistic of their association is shown in the blue-red heatmap (red = positive association, blue = negative association, white = no significant association). Only CpGs which associated with at least 5 genes and genes which associated with at least 2.5% of any clock were included. Genes and CpGs were clustered based on Euclidean distance. Two sidebars (viridis color scale) were included with external data, with the left sidebar depicting expression of *trans*-genes in 17 sorted blood cell types published by Monaco et al. [21], and the top sidebar depicting DNAm of clock CpGs in sorted T-cell and NK-cell subtypes combined from 4 datasets [11,24–26]. External expression and DNAm data were normalized so that each gene/CpG had a DNAm/expression range between 0 and 1 across all measured cell types. The 58 genes and 100 CpGs were compared to the 216 genes and 365 CpGs identified in **Fig. 4**, and 58 genes and 83 CpGs were found to be overlapping.

**
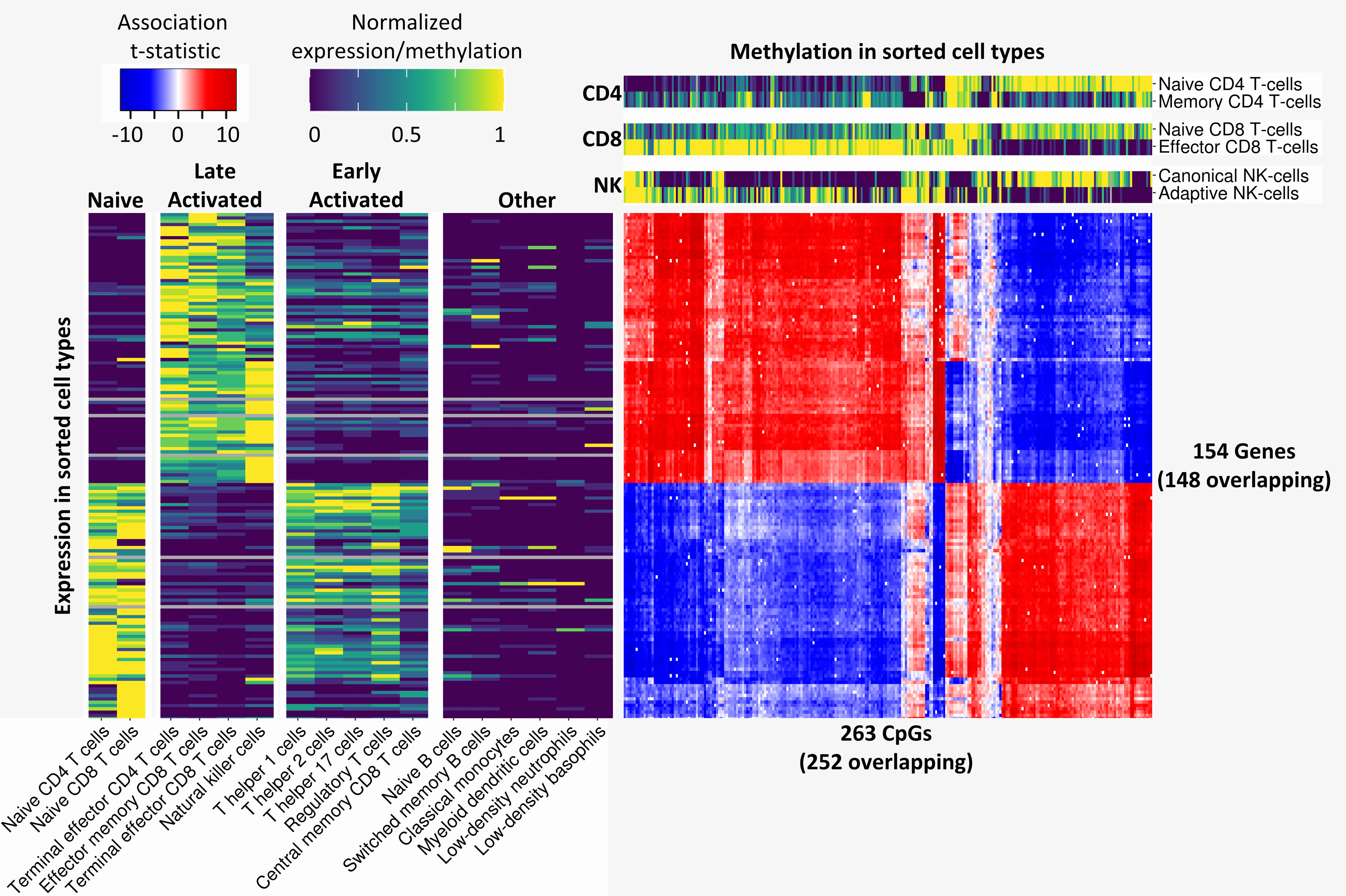
**

**Fig. S5.** Associations between DNAm of clock CpGs and expression of genes *in trans* without predicting white blood cell counts with *wbccpredictor*. This sub-analysis was performed on only the 2,120 samples for which blood cell counts were complete. Per gene-CpG pair, the t-statistic of their association is shown in the blue-red heatmap (red = positive association, blue = negative association, white = no significant association). Only CpGs which associated with at least 10 genes and genes which associated with at least 5% of any clock were included. Genes and CpGs were clustered based on Euclidean distance. Two sidebars (viridis color scale) were included with external data, with the left sidebar depicting expression of *trans*-genes in 17 sorted blood cell types published by Monaco et al. [21], and the top sidebar depicting DNAm of clock CpGs in sorted T-cell and NK-cell subtypes combined from 4 datasets [11,24–26]. External expression and DNAm data were normalized so that each gene/CpG had a DNAm/expression range between 0 and 1 across all measured cell types. The 154 genes and 263 CpGs were compared to the 216 genes and 365 CpGs identified in **Fig. 3**, and 148 genes and 252 CpGs were found to be overlapping.


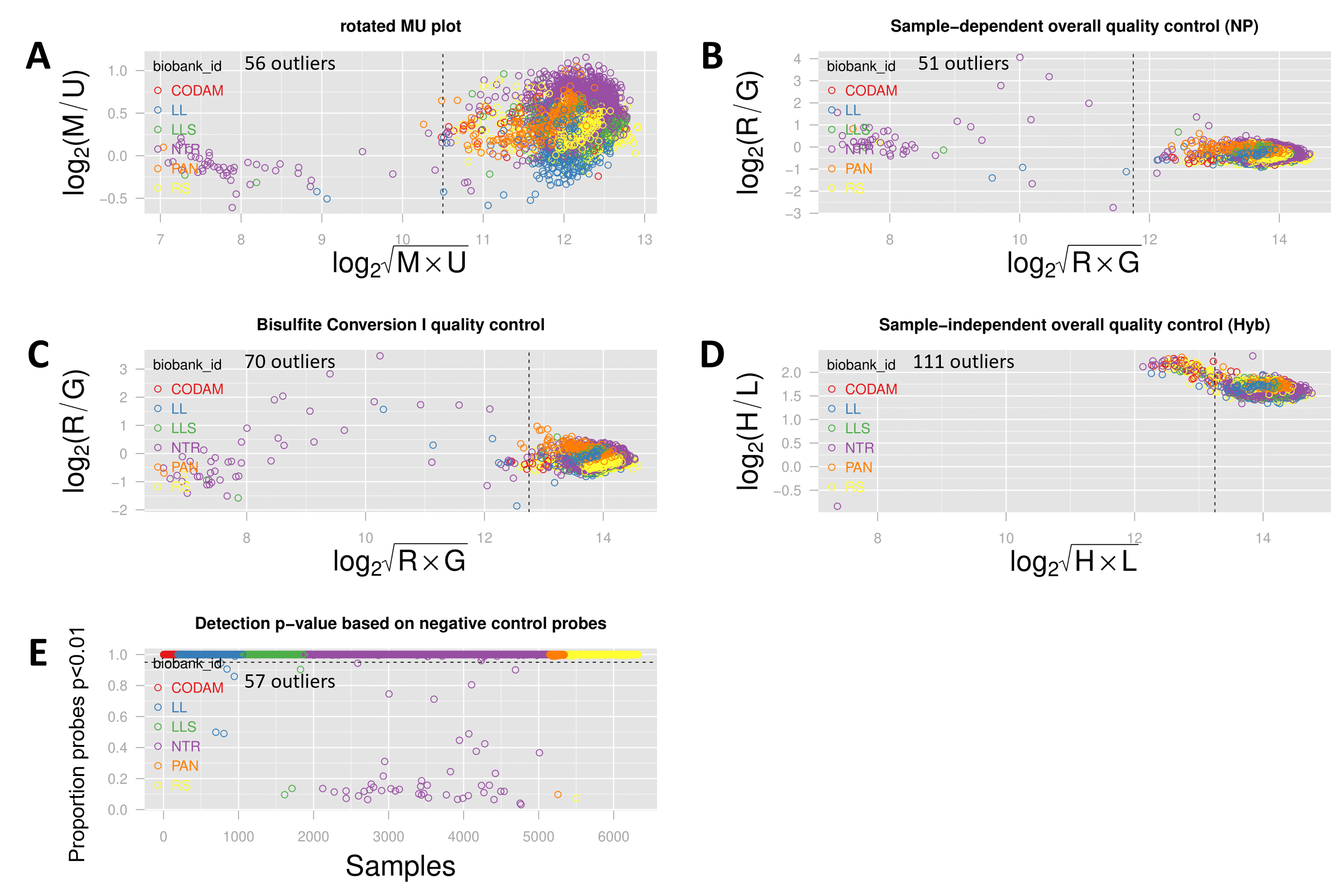


**Fig. S6.** Quality-control (QC) measures for the DNAm data based on control probe information and call rate as determined by the *Methylaid* software. Samples were excluded if they fell outside of at least 1 of the 5 defined QC thresholds, as shown in subfigure **A-E**. Samples were colored for biobank. In each panel, the number of samples falling outside of the threshold is noted. In total, 168 samples were excluded. The used cutoffs used to define outliers were as follows: (**A**) A median Methylated and Unmethylated log2 intensity smaller than 10.5 (rotated MU plot). (**B**) An average log2 intensity of the expected signals in green and red channel of non-polymorphic controls smaller than 11.75 (overall sample-dependent control plot). (**C**) An average log2 intensity of converted Bisulfite Type I controls in green and red channel smaller than 12.75 (bisulfite conversion control plot). (**D**) An average log2 intensity of High and Low hybridization controls (green channel) smaller than 13.25 (overall sample-dependent control plot). (**E**) Less 95% of their probes above the background signal (detection p-value plot).

| **Clock** | **No. Of CpGs** | **ICC according to Sugden et al.** | **ICC in our data** |
| --- | --- | --- | --- |
| Hannum Bld | 71 | 0.29 | 0.78 |
| Horvath MT | 353 | 0.18 | 0.58 |
| Horvath Skn/Bld | 389 | 0.36 | 0.72 |
| Zhang Bld/Slv | 514 | 0.44 | 0.71 |
|  |  |  |  |
| All clocks | 1146 | 0.36 | 0.68 |

**Table S4**. Reliability of the CpGs comprising the 4 investigated epigenetic clocks. Reliability of each clock was quantified using the intraclass correlation (ICC) with a mean-rating (k = 2), absolute-agreement, 2-way random-effects model. ICCs below 0.4 are considered ‘poor’, those between 0.4 and 0.6 are considered ‘fair’, between 0.6 and 0.75 ‘good’, and above 0.75 ‘excellent’. This table shows the median ICC as calculated by Sugden et al., as well as the median ICC as calculated in our own data, using 15 samples for which a technical replicate was available (30 samples in total).

**
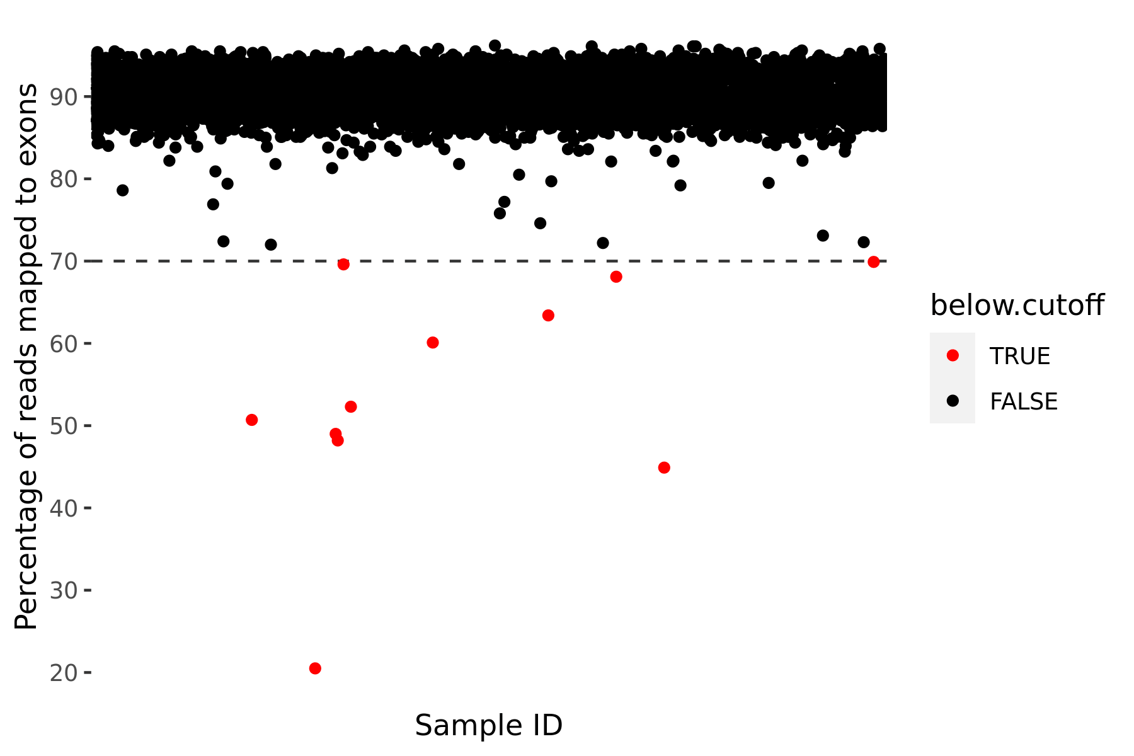
**

**Fig. S7.** Quality-control (QC) measures for the RNA-seq data. Samples were excluded if less than 70% of their reads mapped to exons. In total, 11 samples were excluded.

|  | **Total reads** | **Uniquely mapped reads** | **Uniquely mapped percentage** | **Reads mapped to genome** | **Reads mapped to exons** | **Exon mapped percentage** |
| --- | --- | --- | --- | --- | --- | --- |
| **Mean** | 18796012 | 17147731 | 91.2 | 38558818 | 34724434 | 90.1 |
| **SD** | 4130866 | 3829523 | 2.3 | 8464795 | 7527572 | 2.6 |

**Table S5.** Summary statistics of several QC measures that were gathered during the preprocessing of the RNA-seq data. Total reads is the total number of reads that were gathered for this sample. Uniquely mapped reads is the number of reads which could be mapped to a single spot in the genome (in other words, it excludes ambiguously mapped reads). Uniquely mapped percentage is the percentage of total reads which were uniquely mappable. Reads mapped to genome is the total number of reads mapped to the genome in any place. Reads mapped to exons is the number of reads mapped to exons. Exon mapped percentage is the percentage of all reads that were mapped to exons.
