# Supplementary material for: Functional genomics analysis identifies T and NK cell activation as a driver of epigenetic clock progression
Source: Genome Biol. 2022 Jan 14;23:24. doi: 10.1186/s13059-021-02585-8 (PMC8759260; doi:10.1186/s13059-021-02585-8)
Supplement: Supplementary file 5 — Additional file 5. Review history. [file 13059_2021_2585_MOESM5_ESM.docx]

Review History

**First round of review**

**Reviewer 1**

**Are you able to assess all statistics in the manuscript, including the appropriateness of statistical tests used?**

Yes, and I have assessed the statistics in my report.

**Comments to author:**

Jonkman et al. Provide compelling evidence explaining the function of DNAm based epigenetic clock by shifts of blood cell populations with age going along with a depletion of naïve cells. This is a very important paper putting a scientific ground below the emerging and fascinating developments connected with DNAm clocks. It will help to demystify the field and provide a basis for future progress driven by scientific concepts. In an appropriately revised version, it will be a very strong candidate for publication in Genome Biology.

Major comments:
1) The description of the data analysis is insufficient.
1.1) Provide 4-5 lines at the beginning of the result section about the cohort, the data acquisition, analysis and quality controls.
1.2) The description of 450 data analysis in the Method section is insufficient. It mainly refers the reader to other papers and it does not clarify anything. Where technical replicates prepared and analyzed (this is certainly essential)? How were the fluctuation and reproducibility?
1.3) The biological clocks obviously use only a very tiny fraction of the 450k data. Hence QC for these CpGs is particularly crucial. They should be extracted from the data and information about reproducibility and error margins for these data should be processed and presented.
1.4) I was very distracted by the statement on p. 7 that "missing observations were imputed". This is non-scientific and not acceptable in general unless justified in any specific case. I see no justifications and QC regarding this step. Was the process necessary? What happens with the general story if the fictious data are omitted?
2) p. 2, second but last paragraph and Figure 1D: I did not understand what the authors did in this part and what they want to tell us. It seems trivial that in data sets of many different CpGs some taken from one set will always correlate to some of the other data set. I see no statistics here, no multiple testing correction, and - frankly spoken - as these different CpGs refer to different genomic loci, no scientific rationale for this analysis.
3) Similarly as point 2, provide more details of the results of the QC of the expression data. It is not sufficient to tell the reader which QC steps were conducted, but one needs to show the results to illustrate how reliable data are.
4) The "association" between clock CpGs and transgenes is in the center of the work. It should be described better what is meant by this, how it was determined and it should be documented.
4.1) In my current understanding it refers to a correlation of gene expression and CpG methylation between the different samples. Please clarify. If so, one would expect parameters like R-factor and p-value been discussed.
4.2) Statistical relevance should be confirmed by correlation analysis after randomization of one data set.
4.3) Examples of the correlations should be shown.
4.4) p. 4, line 25-26: What is meant with the statement "genes that were associated with at least 5% of any of the clock"? This sentence does not make sense. 5% of the CpGs of any of the clock? This is stated at other places using a similar phrase. Please use consistent and clear descriptions.
4.5) It should be clarified throughout if the term "association" is used in the meaning of "correlation". If so, "correlation" would be the much preferred term. If not, please explain the difference.
5) Based on the findings of this paper it would be interesting to add a sentence about clocks based on other tissues to the abstract
6) Minor comments:
6.1) p. 2, line 49-51: The model about entropy and 50% is obviously flawed for many reasons and it does not need to be mentioned here. I suggest to change this sentence to "…gradual decline in epigenetic control [9]."
6.2) Fig. 1E: Define the sources of the chromatin data in the legend of the figure. What does "OR" stand for? The column labels are shifted to the left.
6.3) It would be helpful to indicate on the figure, which sidebar shows expression and which methylation.

**Reviewer 2**

**Are you able to assess all statistics in the manuscript, including the appropriateness of statistical tests used?**

Yes, and I have assessed the statistics in my report.

**Comments to author:**

This paper attempts to identify possible mechanisms associated with age dependent DNA methylation changes. They examine CpGs that are included in four different epigenetic clocks, and identify genes whose expression is correlated with these. They identify a set of a few hundred genes that are significantly enriched for T cell activation, and thus conclude that age associated changes in T cell activation may underlie the methylation changes found in epigenetic clocks.

Overall this is a well designed study that leverages a large dataset of joint DNA methylation and gene expression profiles. This data allows the authors to identify genes whose expression is correlated with CpGs that are included in four clocks. The authors also note that most of these associations are in trans and very few in cis. This result could be interpreted in various ways. Are the genes they identify simply age associated genes or are they in some way associated with the specific CpGs that are being tested? If they are simply age associated genes, then the authors have identified a somewhat indirect way of measuring age associated genes, that although of interest, may not explain the underlying DNA methylation changes. Unless there is some hypothesis regarding the association of their Cpg sites with the expression of these genes it is not clear how the observation they present help explain the clocks in a mechanistic fashion. Give the vast set of data on chromatin conformation, is there any way to connect the position of the CpGs with the regulation of these genes? Without such a connection, the observation that T cell activation changes with age is certainly of interest, but does not necessarily explain DNA methylation changes.

===
Please consider the following questions:
- Are the methods appropriate to the aims of the study, are they well described, and are necessary controls included? If not, please specify what is required.
- Are the conclusions adequately supported by the data shown? If not, please explain
- Are sufficient details provided to allow replication and comparison with related analyses that may have been performed? If not, please specify what is required.
- Does the work represent a significant advance over previously published studies?
- Is the paper of broad interest to others in the field, or of outstanding interest to a broad audience of biologists?

**Reviewer 3**

**Are you able to assess all statistics in the manuscript, including the appropriateness of statistical tests used?**

No, I do not feel adequately qualified to assess the statistics.

**Comments to author:**

DNA methylation clocks are compound biomarkers that accurately predict individual age. While there are countless papers describing different applications and adaptations of the original principles, it is still not understood why DNA methylation clocks actually work. The manuscript by Jonkman et al. addresses this very important knowledge gap and finds T- and NK-cell activation as a driver of clock progression. This is highly original and potentially very interesting, but additional controls are required to confirm the validity of key claims, as outlined below.

1.The key claim of T- and NK-cell activation as a driver of clock progression needs further validation to confirm that it represents a genuine conceptual advance. Validation could be achieved by using a cell type decomposition algorithm like EpiScore to show that the proportion of naive/activated T-cells change with age in the same methylation datasets. Another possibility could be to check the evolutionary conservation of the identified mechanism in DNA methylation clocks from other organisms, such as the mouse.

2. DNA methylation is a mechanism that affects gene expression in cis, by modulating transcription factor binding. However, the authors did not observe an association between clock CpGs and gene expression patterns in cis. As the key claim of this paper is to provide a mechanistic explanation of the DNA methylation clock, the authors need to provide a convincing mechanistic explanation for how altered DNA methylation at clock CpGs affects gene expression in trans. Also, if the clock, as the authors claim, is driven mainly by methylation changes associated with the expression of genes which are related to naive/activated T-cells, cis-interactions of CpGs with this specific group of genes need to be analyzed in more detail. Finally, the authors need to rule out that the observed associations are underpinned by genetic variants.

Minor issues:

3. Please run a chromHMM enrichment analysis (or similar) for the CpGs acting in trans, like it was done in figure 1E for all clock CpGs. This may also help addressing the question of the mechanism by which the CpGs which are acting in trans to drive the clock.

4. If there are so many CpGs related to T-cells, there should be a lot of redundancy contained in the clocks. Why are all these CpGs necessary, if they are related to the same targets?

5. Why did the authors leave out the 100kb-5mb range of CpG/gene interaction?

6. For figure 2a, the significance of GO terms is reflected by color, but there is no color bar shown. So it is difficult to estimate how significant these GO terms really are.

**Authors Response**

**Point-by-point responses to the reviewers’ comments:**

Reviewer #1:

Jonkman et al. Provide compelling evidence explaining the function of DNAm based epigenetic clock by shifts of blood cell populations with age going along with a depletion of naïve cells. This is a very important paper putting a scientific ground below the emerging and fascinating developments connected with DNAm clocks. It will help to demystify the field and provide a basis for future progress driven by scientific concepts. In an appropriately revised version, it will be a very strong candidate for publication in Genome Biology.

*We appreciate the reviewer’s encouraging comments.*

Major comments:

1) The description of the data analysis is insufficient.

1.1) Provide 4-5 lines at the beginning of the result section about the cohort, the data acquisition, analysis and quality controls.

*We added the following text at the start of the Results section (p. 2):*

*Our analyses were performed on whole blood samples from 3,132 unrelated individuals, aged 18 to 87, originating from 6 Dutch cohorts (****Table 2****), for which both DNAm data and gene expression data were obtained, measured by Illumina 450K arrays and RNA-seq respectively. Only samples from participants for whom both DNAm and gene expression data passed QC were analyzed.*

| ***Cohort*** | ***Number***  ***of samples*** | ***Age (years)*** | | | ***Male/female***  ***percentage*** |
| --- | --- | --- | --- | --- | --- |
|  |  | ***Mean*** | ***SD*** | ***Range*** |  |
| *CODAM* | *147* | *65.5* | *6.9* | *50-79* | *54%/46%* |
| *LL* | *656* | *46.4* | *13.6* | *18-81* | *43%/57%* |
| *LLS* | *626* | *58.3* | *6.8* | *30-79* | *48%/52%* |
| *NTR* | *854* | *37.8* | *14.8* | *18-79* | *34%/66%* |
| *PAN* | *155* | *67.7* | *9.5* | *37-87* | *61%/39%* |
| *RS* | *694* | *62.5* | *6.3* | *51-87* | *42%/58%* |
| ***Total*** | ***3132*** | ***52.5*** | ***16.2*** | ***18-87*** | ***43%/57%*** |

***Table 2****. Characteristics of the 6 cohorts comprising our dataset.*

1.2) The description of 450 data analysis in the Method section is insufficient. It mainly refers the reader to other papers and it does not clarify anything. Where technical replicates prepared and analyzed (this is certainly essential)? How were the fluctuation and reproducibility?

*We expanded our description of the QC for the DNAm data in the Methods (p. 8) and Figure S6 (p. 28):*

*Quality control (QC), preprocessing and normalization of the data was performed using the Bioconductor R package DNAmArray [48]. A detailed description and R code of the pre-processing workflow, including quality control and normalization, is publicly available at* [*https://molepi.github.io/DNAmArray_workflow/*](https://molepi.github.io/DNAmArray_workflow/)*. In brief, IDAT-files were read into R using minfi [49], after which sample-level QC was performed with MethylAid [50]. Low-quality samples were defined based on 4 QC-measures using information provided by the control probes on the array and 1 measure was based on call rate (>95%) as shown in* ***Figure S6****, which led to the exclusion of 168 samples. Probe-level QC was based on detection p-value (p < 0.01), number of beads available (≤ 2) or zero values for signal intensity. Resulting probes with more than 5% missing values were removed. Normalization was done using functional normalization as implemented in minfi, using five principal components extracted using the control probes for normalization.*

***Figure S6.*** *Quality-control (QC) measures for the DNAm data based on control probe information and call rate as determined by the Methylaid software. Samples were excluded if they fell outside of at least 1 of the 5 defined QC thresholds, as shown in subfigure* ***A****-****E****. Samples were colored for biobank. In each panel, the number of samples falling outside of the threshold is noted. In total, 168 samples were excluded. The used cutoffs used to define outliers were as follows: (****A****) A median Methylated and Unmethylated log2 intensity smaller than 10.5 (rotated MU plot). (****B****) An average log2 intensity of the expected signals in green and red channel of non-polymorphic controls smaller than 11.75 (overall sample-dependent control plot). (****C****) An average log2 intensity of converted Bisulfite Type I controls in green and red channel smaller than 12.75 (bisulfite conversion control plot). (****D****) An average log2 intensity of High and Low hybridization controls (green channel) smaller than 13.25 (overall sample-dependent control plot). (****E****) Less 95% of their probes above the background signal (detection p-value plot).*

*We also included an analysis of technical replicates to get an impression of the reliability of the DNAm data (p. 8-9) and compared our results to those previously reported by Sudgen et al (Patterns 2020):
Sugden et al. analyzed the reliability of measurements of DNAm from Illumina 450K arrays using repeated measurements [53]. First, we downloaded the reported reliability of 450K CpGs as expressed using the intraclass correlation (ICC) and selected the ICC for all CpGs incorporated in the 4 clocks we investigated. The median ICC of clock CpGs was 0.36 (****Table S4****). For context, ICCs below 0.4 were considered ‘poor’, those between 0.4 and 0.6 were considered ‘fair’, between 0.6 and 0.75 ‘good’, and above 0.75 ‘excellent’. Next, we also calculated the ICCs for clock CpGs using our own data on 15 samples for which we had a technical replicate (30 samples in total; ICC was calculated for each CpG using a mean-rating (k = 2), absolute-agreement, and 2-way random-effects model). We found that the reliability of clock CpGs was markedly higher in our data as compared with the analysis of the replicates available to Sugden et al. with median ICC of 0.68, and reaching good or even excellent reliability for most CpGs (****Table S4****). We concluded that the DNAm data we used for in our analysis compare favorably to data used in other reports and can be considered sufficiently reliable.*

| ***DNAm Clock*** | ***No. of CpGs*** | ***ICC according to Sugden et al.*** | ***ICC in our data*** |
| --- | --- | --- | --- |
| *Hannum Bld* | *71* | *0.29* | *0.78* |
| *Horvath MT* | *353* | *0.18* | *0.58* |
| *Horvath Skn/Bld* | *389* | *0.36* | *0.72* |
| *Zhang Bld/Slv* | *514* | *0.44* | *0.71* |
|  |  |  |  |
| *All clocks* | *1146* | *0.36* | *0.68* |

***Table S4****. Reliability of the CpGs comprising the 4 investigated epigenetic clocks as quantified using the intraclass correlation (ICC with a mean-rating (k = 2), absolute-agreement, 2-way random-effects model). ICCs below 0.4 are considered ‘poor’, those between 0.4 and 0.6 are considered ‘fair’, between 0.6 and 0.75 ‘good’, and above 0.75 ‘excellent’. This table shows the median ICC as calculated by Sugden et al., as well as the median ICC as calculated in our own data, using 15 samples for which a technical replicate was available (30 samples in total).*

1.3) The biological clocks obviously use only a very tiny fraction of the 450k data. Hence QC for these CpGs is particularly crucial. They should be extracted from the data and information about reproducibility and error margins for these data should be processed and presented.

*Please refer to our previous answer regarding the analysis of technical replicates (comment 1.2).*

1.4) I was very distracted by the statement on p. 7 that "missing observations were imputed". This is non-scientific and not acceptable in general unless justified in any specific case. I see no justifications and QC regarding this step. Was the process necessary? What happens with the general story if the fictious data are omitted?

*To evaluate whether imputation of missing data influenced our results, we repeated our analysis using the subset of 2120 samples for which we had complete measured cell count. Although this analysis included fewer samples and hence had a lower statistical power, the results were highly comparable (p.6 for text, p. 27 for figure). This sensitivity analysis indicates that the procedure of imputation did not influence the results or conclusions of our analysis. New text and figures:*

*Additionally, to check the validity of our method of predicting main blood cell counts, we repeated the analysis with only the 2,120 samples for which cell counts were measured, now using measured cell counts as covariates. The results from this analysis were very similar to the original analysis using predicted cell counts (****Figure 4****). Adopting the same cutoffs as for the original analysis (genes which associate with at least 5% of any clock and CpGs associating with 10 genes), we identified 263 clock CpGs associating with trans-154 genes (****Figure S5****). This a lower number than the original analysis, likely due to the lower sample size, but the sets were very similar: 252 out of 263 CpGs and 148 out of the 154 genes were also identified in the original analysis with the complete set of 3,132 samples. Also, the genes and CpGs formed the same two clusters representing naive and activated immune phenotypes (****Figure S5****, left and top sidebars). Therefore, the prediction of main cell counts in order to analyze the complete set of samples has not influenced our findings.*

***Figure S5.*** *Associations between DNAm of clock CpGs and expression of genes in trans without predicting white blood cell counts with wbccpredictor. This sub-analysis was performed on only the 2120 samples for which blood cell counts were complete. Per gene-CpG pair, the t-statistic of their association is shown in the blue-red heatmap (red = positive association, blue = negative association, white = no significant association). Only CpGs which associated with at least 10 genes and genes which associated with at least 5% of any clock were included. Genes and CpGs were clustered based on Euclidean distance. Two sidebars (viridis color scale) were included with external data, with the left sidebar depicting expression of trans-genes in 17 sorted blood cell types published by Monaco et al. [21], and the top sidebar depicting DNAm of clock CpGs in sorted T-cell and NK-cell subtypes published by Garaud et al. [11] and Schlums et al. [22]. External expression and DNAm data were normalized so that each gene/CpG had a DNAm/expression range between 0 and 1 across all measured cell types. The 154 genes and 263 CpGs were compared to the 216 genes and 365 CpGs identified in Figure 3, and 148 genes and 252 CpGs were found to be overlapping.*

2) p. 2, second but last paragraph and Figure 1D: I did not understand what the authors did in this part and what they want to tell us. It seems trivial that in data sets of many different CpGs some taken from one set will always correlate to some of the other data set. I see no statistics here, no multiple testing correction, and - frankly spoken - as these different CpGs refer to different genomic loci, no scientific rationale for this analysis.

*We agree that Figure 1D and the accompanying section in the text were not sufficiently clear. We re-designed Figure 1D using a different approach which we think is more intuitive (p. 17), and also re-wrote the text (p. 3):*

*“Notably, the similar predictions of the 4 clocks may not only stem from CpGs shared between the clocks, but also from non-shared CpGs whose methylation levels are correlated. Therefore, we analyzed, per clock, whether each of its CpGs had at least one moderate, strong, or very strong proxy (defined as absolute correlation above 0.5, 0.7, and 0.9 respectively) in the 3 other clocks. The majority of clock CpGs had at least a moderate proxy in the other clocks (****Figure 1D****); the percentages varied from 94% (Hannum Bld with Horvath Skn/Bld) to 45% (Horvath MT with Hannum Bld). Therefore, it is plausible that both overlapping CpGs and correlation between non-overlapping CpGs contributed to the similar performance of the 4 clocks in predicting chronological age. Interestingly, while all epigenetic were trained using elastic net, a penalized regression method to limit correlation between features in a predictor [2–5], we nevertheless observed fairly strong internal correlations within each clock, with 45% to 96% of the clocks’ CpGs having a moderate proxy within the same clock (****Figure 1D****). This indicates that there is notable redundancy of the CpGs included in the clocks.”*

***Figure 1.*** *Characteristics of epigenetic clocks. (****A****) Prediction of age by the four epigenetic clocks. Prediction accuracy was analyzed using Pearson correlation (r) between chronological and predicted age, as well as mean absolute error (MAE) of the predicted ages. (****B****) Concordance of the age prediction errors of the epigenetic clocks, analyzed using Pearson correlation (r) between the prediction errors of each clock pair. (****C****) Overlap of the CpGs comprising the epigenetic clocks. The cells on the diagonal represent total clock size, while the other cells represent the number of CpGs shared between each clock pair. The color of each cell represents the percentage of the smaller CpG set that overlaps with the larger CpG set (0% = white, 100% = red). (****D****) Percentage of each clock’s CpGs which have a proxy (defined by absolute Pearson correlation above 0.5) within the same clock or in the 3 other clocks. Percentages with a proxy correlation of at least 0.5, 0.7, and 0.9 are plotted in stacked bar charts. Correlations within a clock are marked with an asterisk (*). (****E****) Enrichment of clock CpGs in CpG-island-centric features (top section), histone modifications (middle section), and chromatin states according to ChromHmm (bottom section). (****Abbreviations****) OR: odds ratio; CGI: CpG island; TssA: Active transcription start site; TssAFlnk: flanking active transcription start site; TxFlnk: transcription at gene 5' and 3'; Tx: strong transcription; TxWk: weak transcription; EnhG: genic enhancers; Enh: enhancers; ZNF/Rpts: ZNF genes plus repeats; Het: heterochromatin; TssBiv: bivalent/poised transcription start site; BivFlnk: flanking bivalent transcription start site/enhancer; EnhBiv: bivalent enhancer; ReprPC: repressed polycomb; ReprPCWk: weak repressed polycomb; Quies: quiescent/low.*

*Furthermore, we now describe the rationale of this analysis in the Methods (p. 10):*

*“To obtain an impression of the degree to which the CpGs comprising the 4 clocks capture the same information, we analyzed the correlation of their methylation values. The clocks were trained using elastic net, a penalized regression method which aims to minimize internal correlations within a predictor. Therefore, it is expected that most correlations in DNA methylation of CpGs within and between clocks are (very) low. Therefore, we reported the percentage of each clock’s CpGs which had a correlation with another CpG’s methylation level of at least 0.5, 0.7, and 0.9 within the same clock and in the 3 other clocks. For correlations between clocks, instances where the same CpG was included in two clocks were included as a correlation of 1; for correlations within a clock, the correlation of the CpGs with itself was excluded.”*

3) Similarly as point 2, provide more details of the results of the QC of the expression data. It is not sufficient to tell the reader which QC steps were conducted, but one needs to show the results to illustrate how reliable data are.

*We have described the QC measure based on which we selected RNA-seq samples in the Methods (p. 9) and Figure S7 (p. 28):*

*“Samples for which less than 70% of all reads mapped to exons were removed, as shown in* ***Figure S7****; this was the case for 11 samples.”*

***Figure S7.*** *Quality-control (QC) measures for the RNA-seq data. Samples were excluded if less than 70% of their reads mapped to exons. In total, 11 samples were excluded.*

*Furthermore, we now also provide summary data regarding the RNA-seq samples that passed QC in supplementary Table S5:*

|  | ***Total reads*** | ***Uniquely mapped reads*** | ***Uniquely mapped percentage*** | ***Reads mapped to genome*** | ***Reads mapped to exons*** | ***Exon mapped percentage*** |
| --- | --- | --- | --- | --- | --- | --- |
| ***Mean*** | *18796012* | *17147731* | *91.2* | *38558818* | *34724434* | *90.1* |
| ***SD*** | *4130866* | *3829523* | *2.3* | *8464795* | *7527572* | *2.6* |

***Table S5****. QC measures that were gathered during the pre-processing of the RNA-seq data. Total reads is the total number of reads that were gathered for this sample. Uniquely mapped reads is the number of reads which could be mapped to a single spot in the genome (in other words, it excludes ambiguously mapped reads). Uniquely mapped percentage is the percentage of total reads which were uniquely mappable. Reads mapped to genome is the total number of reads mapped to the genome in any place. Reads mapped to exons is the number of reads mapped to exons. Exon mapped percentage is the percentage of reads mapped to exons.*

4) The "association" between clock CpGs and transgenes is in the center of the work. It should be described better what is meant by this, how it was determined and it should be documented.

*Indeed, the reviewer is correct that the association or correlation is the center of the work. And we now also see how this could lead to misunderstanding by readers who may incorrectly assume that we imply a mechanistic link. In this and the following points we address this issue and in 4.5 propose textual changes to make this point more explicit.*

*We prefer the term association because the term correlation may imply that we report on Pearson or any other type of correlation, whereas we performed a linear regression analysis for each gene-CpG pair and selected genes and CpGs based on regression p-values, testing whether the beta of the methylation in the statistical model is unequal to 0 after accounting for a range of covariates, inflation of test statistics, and correction for multiple testing (Bonferroni). Of course, association has the same meaning as correlation in the sense that no causation is implied. Also, we do see strong correlations for example between the trans-genes and CpGs (see 4.3).*

4.1) In my current understanding it refers to a correlation of gene expression and CpG methylation between the different samples. Please clarify. If so, one would expect parameters like R-factor and p-value been discussed.

*See the explanation on comment 4.*

4.2) Statistical relevance should be confirmed by correlation analysis after randomization of one data set.

*We randomized the subject identifiers of the DNAm-data 1,000 times, then re-ran our regression analysis for each permutation. This analysis confirmed the statistical relevance of our results, as described in the Methods (p. 11).*

*To confirm the validity of our analysis, we performed a permutation test where we randomized the sample identifiers of the DNAm data 1,000 times, then repeated the analysis and counted the total number of Bonferroni-significant trans-associations. Our actual analysis identified 50,029 associations, while all 1,000 permutations identified 0 associations (p < 0.001).*

*We also mention the permutation test in the Results (p. 4).*

*A permutation test confirmed the statistical significance of association between CpGs and trans-genes (p < 0.001).*

4.3) Examples of the correlations should be shown.

*This is an excellent idea. We added a figure containing examples of positive and negative associations in Figure 3 (p. 19), and believe that these figures may help the readers to better follow our findings. The examples are of the trans-genes that we already mentioned in the text. We included the correlation here, as requested by the reviewer:*

***Figure 3****. Examples of positive and negative correlations between clock CpG DNAm and gene expression (rank-inverse normal transformed), as identified in the linear regression analysis. The subfigures show the Pearson correlation (r) between gene expression and DNA-methylation for a gene-CpG pair, as well as the p-value for the correlation. (****A****) Positive and negative correlations identified for three genes with known expression in naïve T-cells: FOXP1, LEF1, and TMIGD2. (****B****) Positive and negative correlations identified for three genes with known expression in activated T-cells: LAG3, EOMES, and GZMH.*

4.4) p. 4, line 25-26: What is meant with the statement "genes that were associated with at least 5% of any of the clock"? This sentence does not make sense. 5% of the CpGs of any of the clock? This is stated at other places using a similar phrase. Please use consistent and clear descriptions.

*Thank you for pointing this out. We have added a clarifying sentence to the Results (p. 4):*

*“We hypothesized that the trans-genes that provide information on the biological phenomena driving changes in DNAm would be associated with multiple CpGs and vice versa. Therefore, we focused our further analysis on a core set of 216 trans-genes that were associated with at least 5% of any of the 4 investigated clocks’ CpGs (in other words, genes which associated with either 4 Hannum Bld CpGs, 18 Horvath MT CpGs, 20 Horvath Skn/Bld CpGs, or 26 Zhang Bld/Slv CpGs), and a core set of 365 CpGs that associated with at least 10 trans-genes.”*

*The same information was added to the Methods (p. 11):*

*“For the trans-associations, a core set of genes and CpGs was selected. We selected genes which were significantly associated (p_Bonf_ < 0.05) with at least 5% of the CpGs comprising any of the 4 investigated clocks (in other words, genes which associated with either 4 Hannum Bld CpGs, 18 Horvath MT CpGs, 20 Horvath Skn/Bld CpGs, or 26 Zhang Bld/Slv CpGs), and we selected CpGs which were significantly associated with at least 10 genes.”*

4.5) It should be clarified throughout if the term "association" is used in the meaning of "correlation". If so, "correlation" would be the much preferred term. If not, please explain the difference.

*We see the point of the reviewer. What we look for are indeed correlative markers of the underlying phenomena and not mechanisms; we do not assume or search for a direct mechanistic link between DNA methylation and trans-genes. As mentioned earlier, we prefer the term association because correlation may indicate to the reader that we performed a correlation analysis, while we actually ran multivariate regression models accounting for measured and unmeasured confounders, corrected for bias and inflation, and applied a proper correction for multiple testing.*

*We now clarified our reasoning stating that we look for correlation/markers and how we implemented that by testing for associations on p. 3.*

*“To gain more insight into the biological phenomena that are captured by the epigenetic clocks, we investigated how DNAm of the individual clock CpGs correlated with gene expression. We did this by performing a linear regression analysis to find the associations between clock CpG DNAm and expression of genes in cis and in trans.”*

*We also clarify this point further in the Discussion (p. 7).*

*“It should be noted that our aim was not to find or imply any direct causal link between clock CpG DNAm and trans-gene expression. Instead, we characterized clock CpGs by analyzing their correlation with trans-gene expression. The inspection of the function and cell-specific expression of these trans-genes subsequently led to the interpretation that the trans-genes and the correlating clock CpGs were both markers of the same blood cell type proportions, namely that between naive and activated T- and NK-cells. Hence, our data support the interpretation that age-related changes in clock CpG methylation and trans-gene expression have shifts in blood cell types as a common cause, which leads to a notable correlation between the two.”*

5) Based on the findings of this paper it would be interesting to add a sentence about clocks based on other tissues to the abstract

*We agree that this is an interesting finding and mention it in the Discussion. Unfortunately, we already are at the maximum word count for our Abstract*.

6) Minor comments:

6.1) p. 2, line 49-51: The model about entropy and 50% is obviously flawed for many reasons and it does not need to be mentioned here. I suggest to change this sentence to "…gradual decline in epigenetic control [9]."

*We agree and have edited this sentence (p. 2).*

*“The chronological age as predicted by Horvath’s MT clock [3] was shown to be independent from the accumulation of senescent cells [8] and the progression this clock has been hypothesized to be due to a gradual decline in epigenetic control [9].”*

6.2) Fig. 1E: Define the sources of the chromatin data in the legend of the figure. What does "OR" stand for? The column labels are shifted to the left.

*Thanks for noting this. We fixed the figure (p. 17).*

***Figure 1.*** *Characteristics of epigenetic clocks. (****A****) Prediction of age by the four epigenetic clocks. Prediction accuracy was analyzed using Pearson correlation (r) between chronological and predicted age, as well as mean absolute error (MAE) of the predicted ages. (****B****) Concordance of the age prediction errors of the epigenetic clocks, analyzed using Pearson correlation (r) between the prediction errors of each clock pair. (****C****) Overlap of the CpGs comprising the epigenetic clocks. The cells on the diagonal represent total clock size, while the other cells represent the number of CpGs shared between each clock pair. The color of each cell represents the percentage of the smaller CpG set that overlaps with the larger CpG set (0% = white, 100% = red). (****D****) Percentage of each clock’s CpGs which have a proxy (defined by absolute Pearson correlation above 0.5) within the same clock or in the 3 other clocks. Percentages with a proxy correlation of at least 0.5, 0.7, and 0.9 are plotted in stacked bar charts. Correlations within a clock are marked with an asterisk (*). (****E****) Enrichment of clock CpGs in CpG-island-centric features (top section), histone modifications (middle section), and chromatin states according to ChromHmm (bottom section). (****Abbreviations****) OR: odds ratio; CGI: CpG island; TssA: Active transcription start site; TssAFlnk: flanking active transcription start site; TxFlnk: transcription at gene 5' and 3'; Tx: strong transcription; TxWk: weak transcription; EnhG: genic enhancers; Enh: enhancers; ZNF/Rpts: ZNF genes plus repeats; Het: heterochromatin; TssBiv: bivalent/poised transcription start site; BivFlnk: flanking bivalent transcription start site/enhancer; EnhBiv: bivalent enhancer; ReprPC: repressed polycomb; ReprPCWk: weak repressed polycomb; Quies: quiescent/low.*

6.3) It would be helpful to indicate on the figure, which sidebar shows expression and which methylation.

*This information has been added to all heatmap figures that include expression and methylation in sorted cell types (Figure 4, S4, and S5). Figure 3 is shown here.*

***Figure 4.*** *DNAm of clock CpGs and expression of trans-genes across sorted blood cell types. Per gene-CpG pair, the t-statistic of their association is shown in the blue-red heatmap (red = positive association, blue = negative association, white = no significant association). Only CpGs which associated with at least 10 genes and genes which associated with at least 5% of any clock were included. Genes and CpGs were clustered based on Euclidean distance. Two sidebars (viridis color scale) were included with external data, with the left sidebar depicting expression of trans-genes in 17 sorted blood cell types published by Monaco et al. [21], and the top sidebar depicting DNAm of clock CpGs in sorted T-cell and NK-cell subtypes combined from 4 datasets [11,24–26]. External expression and DNAm data were normalized so that each gene/CpG had a DNAm/expression range between 0 and 1 across all measured cell types.*

Reviewer #2:

This paper attempts to identify possible mechanisms associated with age dependent DNA methylation changes. They examine CpGs that are included in four different epigenetic clocks, and identify genes whose expression is correlated with these. They identify a set of a few hundred genes that are significantly enriched for T cell activation, and thus conclude that age associated changes in T cell activation may underlie the methylation changes found in epigenetic clocks.

Overall this is a well designed study that leverages a large dataset of joint DNA methylation and gene expression profiles. This data allows the authors to identify genes whose expression is correlated with CpGs that are included in four clocks. The authors also note that most of these associations are in trans and very few in cis. This result could be interpreted in various ways. Are the genes they identify simply age associated genes or are they in some way associated with the specific CpGs that are being tested? If they are simply age associated genes, then the authors have identified a somewhat indirect way of measuring age associated genes, that although of interest, may not explain the underlying DNA methylation changes. Unless there is some hypothesis regarding the association of their Cpg sites with the expression of these genes it is not clear how the observation they present help explain the clocks in a mechanistic fashion. Give the vast set of data on chromatin conformation, is there any way to connect the position of the CpGs with the regulation of these genes? Without such a connection, the observation that T cell activation changes with age is certainly of interest, but does not necessarily explain DNA methylation changes.

*We have clarified our approach and interpretation in the text (also in line with question of reviewer 1). In this study, we do not look for direct mechanistic links between the CpGs and the trans-genes. Rather, we use the trans-genes we identified as a correlate of the phenomena that underlie running of the DNAm clocks. Indeed, the trans-genes point to shifts in T cell subtypes with age (from naïve to activated) as the drivers of the clocks. The association between clock CpG levels and trans-genes thus stems from the fact that both change when proportions of T- and NK-cell subtypes change.*

*We now clarified our reasoning stating that we look for correlation/markers and how we implemented that by testing for associations on p. 3.*

*“To gain more insight into the biological phenomena that are captured by the epigenetic clocks, we investigated how DNAm of the individual clock CpGs correlated with gene expression. We did this by performing a linear regression analysis to find the associations between clock CpG DNAm and expression of genes in cis and in trans. “*

*We also clarify this point further in the Discussion (p. 7).*

*“It should be noted that our aim was not to find or imply any direct causal link between clock CpG DNAm and trans-gene expression. Instead, we characterized clock CpGs by analyzing their correlation with trans-gene expression. The inspection of the function and cell-specific expression of these trans-genes subsequently led to the interpretation that the trans-genes and the correlating clock CpGs were both markers of the same blood cell type proportions, namely that between naive and activated T- and NK-cells. Hence, our data support the interpretation that age-related changes in clock CpG methylation and trans-gene expression have shifts in blood cell types as a common cause, which leads to correlation between the two.”*

Reviewer #3:

DNA methylation clocks are compound biomarkers that accurately predict individual age. While there are countless papers describing different applications and adaptations of the original principles, it is still not understood why DNA methylation clocks actually work. The manuscript by Jonkman et al. addresses this very important knowledge gap and finds T- and NK-cell activation as a driver of clock progression. This is highly original and potentially very interesting, but additional controls are required to confirm the validity of key claims, as outlined below.

1.The key claim of T- and NK-cell activation as a driver of clock progression needs further validation to confirm that it represents a genuine conceptual advance. Validation could be achieved by using a cell type decomposition algorithm like EpiScore to show that the proportion of naive/activated T-cells change with age in the same methylation datasets. Another possibility could be to check the evolutionary conservation of the identified mechanism in DNA methylation clocks from other organisms, such as the mouse.

*We agree that this is indeed an interesting question. A complicating factor for our use case, however, is the fact that we are interested in the proportion of specific cell subtypes (i.e. naïve and activated T- and NK-cells). This means that most deconvolution methods are not suitable for us, since they estimate more broad cell types (e.g. all CD8^+^ T cells). One deconvolution method that does estimate these specific cell subtype proportions was the algorithm developed by Monaco et al. (Cell Reports 2019). We implemented this deconvolution method and applied it to our data to accommodate the reviewer’s request. The algorithm confirmed that naive CD8^+^ T cell proportions are reduced with age (R = -0.25, p < 10^-15^) while memory CD8^+^ T cell increase (R = 0.15, p < 10^-15^) with age. However, we are concerned that the deconvolution method is not reliable. Surprisingly, many of the estimated T cell proportions were actually negative (for instance, estimated naïve CD8^+^ T cell proportions ranged from -1% to -28%), which obviously is not possible. This prompted us to compare monocyte counts as predicted by Monaco et al.’s algorithm with our own measured monocyte counts. The two were correlated very weakly with a R of only 0.16 (see scatterplot below). All in all, although we agree that prediction of T cell subtypes and show their association with age could be of interest, prediction of these subsets is not possible in a reliable way at this point. As stated in the Discussion, we think that direct measurement of T cell subsets (e.g. using flow cytometry) may be an interesting way forward in future research.*

***Figure x*** *(not included in manuscript)****.*** *Correlation between monocyte counts in as measured via flow cytometry and monocyte counts as predicted by the deconvolution algorithm developed by Monaco et al.*

2. DNA methylation is a mechanism that affects gene expression in cis, by modulating transcription factor binding. However, the authors did not observe an association between clock CpGs and gene expression patterns in cis. As the key claim of this paper is to provide a mechanistic explanation of the DNA methylation clock, the authors need to provide a convincing mechanistic explanation for how altered DNA methylation at clock CpGs affects gene expression in trans.

*In our study we did not look for or imply direct causal or mechanistic links between clock CpG methylation and trans-gene expression. Rather, we used the association with gene expression as a way to understand the biological context in which clock CpGs gain or lose methylation. In other words, we consider clock CpG methylation and trans-gene expression to be indicative of the same underlying shifts in cell proportions, which explains their statistical association. It would be interesting for future work to investigate the precise molecular mechanisms between clock CpGs and trans-genes.*

*We now clarified our reasoning stating that we look for correlation/markers and how we implemented that by testing for associations on p. 3.*

*“To gain more insight into the biological phenomena that are captured by the epigenetic clocks, we investigated how DNAm of the individual clock CpGs correlated with gene expression. We did this by performing a linear regression analysis to find the associations between clock CpG DNAm and expression of genes in cis and in trans.”*

*We also clarify this point further in the Discussion (p. 7).*

*“It should be noted that our aim was not to find or imply any direct causal link between clock CpG DNAm and trans-gene expression. Instead, we characterized clock CpGs by analyzing their correlation with trans-gene expression. The inspection of the function and cell-specific expression of these trans-genes subsequently led to the interpretation that the trans-genes and the correlating clock CpGs were both markers of the same blood cell type proportions, namely that between naive and activated T- and NK-cells. Hence, our data support the interpretation that age-related changes in clock CpG methylation and trans-gene expression have shifts in blood cell types as a common cause, which leads to correlation between the two.”*

Also, if the clock, as the authors claim, is driven mainly by methylation changes associated with the expression of genes which are related to naive/activated T-cells, cis-interactions of CpGs with this specific group of genes need to be analyzed in more detail.

*We use gene expression data as correlative marker of the phenomena underlying DNAm clocks. Our analysis indicates that shifts in T- and NK-cell subset abundance are a key aspect. Clock CpG methylation levels and trans-gene expression are correlated because they both change with changes in immune cell composition. Hence, the methylation changes and expression changes have immune cell changes a common cause leading to correlation between the two. However, this does not imply or necessitates, and we also do not claim, that there is a mechanistic link between the two in a molecular way. To accommodate the reviewer’s criticisms, we manually inspected individual cis-genes for their biological function because the gene-set is too small for a meaningful gene ontology analysis. No overarching patterns in gene function could be identified. We now reported this finding in more detail (p. 3-4):*

*“For none of the clocks, the genes associating in cis were enriched for biological processes according to the gene ontology (GO) database, and no overarching patterns were identified upon manual inspection of the cis-genes individually.”*

Finally, the authors need to rule out that the observed associations are underpinned by genetic variants.

*To rule out genetic effects, we have compared two recently published resources, the first containing methylation-QTLs (Min et al., Nature Genetics 2021), and the second containing expression-QTLs (Võsa et al., Nature Genetics 2021).*

*We describe this analysis in the Results (p. 5):*

*“To rule out that the observed trans-associations were caused by genetic variants, we compared two recently published quantitative trait locus (QTL) resources, the first containing methylation(me)-QTLs [1], and the second containing expression(e)-QTLs [1]. We reasoned that a trans-association driven by a genetic variant would require the variant in question to be simultaneously a cis-meQTL for the CpG and a trans-eQTL for the gene, or vice versa. We tested, for each clock CpG identified in the regression analysis, whether it had a known cis-meQTL. Then we tested whether the identified cis-meQTLs (if any) were also identified as trans-eQTL for the genes that significantly associated with that CpG. Only 9 out of 365 CpGs shared one or more SNP with a total of 31 trans-genes (****Table S3****); the 31 trans-genes were also associated with 339 other CpGs for which no QTL effect was found. We also performed this analysis in the opposite direction (testing for each trans-gene if it is driven by cis-eQTLs which are also trans-meQTLs for any of the CpGs that significantly associated with the gene), and found that only 2 out of the 216 trans-genes shared a SNP with 2 clock CpGs. We conclude that genetic effects cannot explain our observations.”*

Minor issues:

3. Please run a chromHMM enrichment analysis (or similar) for the CpGs acting in trans, like it was done in figure 1E for all clock CpGs. This may also help addressing the question of the mechanism by which the CpGs which are acting in trans to drive the clock.

*We attempted to do this. A limitation of such an enrichment analysis is that it cannot reliably identify enrichments if the dataset becomes too small. We already had relatively few CpGs (1,146 total, which is less than 1% of the total 450K array) in our original analysis (Figure 1E, p. 17), which made it so that many annotations had very few CpGs mapped to it, making enrichment calculation for these annotations impossible. This problem is further exacerbated if we select only the 365 CpGs acting in trans, so we find very few significant enrichments, not because of biological meaning, but because of the limitations of the methods. The figure below illustrates this limitation and we prefer not to include the results of this analysis in the manuscript.*

***Figure x*** *(not included in manuscript)****.*** *Statistical enrichments for genomic annotations of the 365 CpGs which associate with gene expression in trans.*

4. If there are so many CpGs related to T-cells, there should be a lot of redundancy contained in the clocks. Why are all these CpGs necessary, if they are related to the same targets?

*The original clocks were trained using elastic net procedures which indeed allow for correlation between CpGs within a clock. Indeed, we observe substantial correlation between CpGs within a clock. We have included this finding in Figure 1D (p. 17) and also briefly describe it in the Results (p. 3). So, indeed there is redundancy within the clocks as already presumed by the reviewer. We feel that a discussion of how the clocks were developed, the mathematics of elastic net, and how this approach influences the CpGs selected for the DNAm clocks is beyond the scope of our current paper. We take the clocks as they are widely used in the literature without having an opinion on the procedures used to develop them.*

*Interestingly, while all epigenetic were trained using elastic net, a penalized regression method to limit correlation between features in a predictor [2–5], we nevertheless observed fairly strong internal correlations within each clock, with 45% to 96% of the clocks’ CpGs having a moderate proxy within the same clock (****Figure 1D****). This indicates that there is notable redundancy of the CpGs included in the clocks.*

***Figure 1.*** *Characteristics of epigenetic clocks. (****A****) Prediction of age by the four epigenetic clocks. Prediction accuracy was analyzed using Pearson correlation (r) between chronological and predicted age, as well as mean absolute error (MAE) of the predicted ages. (****B****) Concordance of the age prediction errors of the epigenetic clocks, analyzed using Pearson correlation (r) between the prediction errors of each clock pair. (****C****) Overlap of the CpGs comprising the epigenetic clocks. The cells on the diagonal represent total clock size, while the other cells represent the number of CpGs shared between each clock pair. The color of each cell represents the percentage of the smaller CpG set that overlaps with the larger CpG set (0% = white, 100% = red). (****D****) Percentage of each clock’s CpGs which have a proxy (defined by absolute Pearson correlation above 0.5) within the same clock or in the 3 other clocks. Percentages with a proxy correlation of at least 0.5, 0.7, and 0.9 are plotted in stacked bar charts. Correlations within a clock are marked with an asterisk (*). (****E****) Enrichment of clock CpGs in CpG-island-centric features (top section), histone modifications (middle section), and chromatin states according to ChromHmm (bottom section). (****Abbreviations****) OR: odds ratio; CGI: CpG island; TssA: Active transcription start site; TssAFlnk: flanking active transcription start site; TxFlnk: transcription at gene 5' and 3'; Tx: strong transcription; TxWk: weak transcription; EnhG: genic enhancers; Enh: enhancers; ZNF/Rpts: ZNF genes plus repeats; Het: heterochromatin; TssBiv: bivalent/poised transcription start site; BivFlnk: flanking bivalent transcription start site/enhancer; EnhBiv: bivalent enhancer; ReprPC: repressed polycomb; ReprPCWk: weak repressed polycomb; Quies: quiescent/low.*

5. Why did the authors leave out the 100kb-5mb range of CpG/gene interaction?

*We agree that the exact limits that are generally being used to distinguish cis- and trans-effects in bioinformatics analyses are fairly arbitrary. We chose the limit of 100kb as a somewhat conservative distance to ensure that cis-associations are genuinely in cis, and similarly we chose 5MB as a conservative distance to ensure that trans-associations are truly in trans.*

6. For figure 2a, the significance of GO terms is reflected by color, but there is no color bar shown. So it is difficult to estimate how significant these GO terms really are.

*Thanks for spotting this. We added the color bar.*

***Figure 2.*** *Associations between clock CpG DNAm and gene expression. (****A****) Percentages of CpGs belonging to the clocks which associate with the expression of at least 1 gene in cis (<100 kB distance). (****B****) Overlap of the genes associating with clock CpGs in cis. The cells on the diagonal represent the total number of genes associating with the CpGs of the clock in cis, while the other cells represent the number of cis-genes shared between each clock pair. The color of each cell represents the percentage of the smaller gene set that overlaps with the larger gene set (0% = white, 100% = red). (****C****) Percentages of CpGs belonging to the clocks which associate with the expression of at least 1, 10, or 100 genes in trans (>5 MB distance or different chromosomes). (****D****) Overlap of the genes associating with clock CpGs in trans. The cells on the diagonal represent the total number of genes associating with the CpGs of the clock in trans, while the other cells represent the number of trans-genes shared between each clock pair. The color of each cell represents the percentage of the smaller gene set that overlaps with the larger gene set (0% = white, 100% = red). Only trans-genes which were associated with at least 5% of any of the clocks were included. (****E****) Biological pathway gene ontology (GO) enrichments of trans-genes. Only trans-genes which were associated with at least 5% of any of the clocks were included. Networks were created using REVIGO, filtering out extremely similar GO-terms for clarity [54]. Nodes represent GO-terms, with node size depicting GO-term generality, and node color depicting enrichment p-value. Highly related GO-terms which pass the similarity filter are connected by edges in the graph, with edge width representing term similarity.*

**Second round of review**

**Reviewer 1**

My questions and comments were convincingly taken up and answered.

**Reviewer 2**

The authors have adequately addressed my concerns

**Reviewer 3**

The authors have streamlined the manuscript and appear to have fixed many technical issues. The focus on trans genes provides for a well-rounded and interesting story. However, the absence of any meaningful cis genes also curtails the conceptual potential of the study considerably because it eliminates any potential mechanistic insight. As the manuscript does not contain any functional or mechanistic insight, T-cell activation should not be described as a "driver" and this needs to be toned down in the title and throughout the text. The authors should add a paragraph to the discussion that explains the (potential) biological role of T-cells and NK-cells in aging.
